# Bioinspired Cold‐Laminated Ultrathin Hydrogels as a Broadly Adaptive Platform for Physiological Monitoring

**DOI:** 10.1002/advs.202524317

**Published:** 2026-02-15

**Authors:** Hui Chen, Jian Zhou, Jianfei Xie, Lin Shi, Lu Wang, Yuanfan Yang, Yihao Guo, Jianhui Cao, Qingyun Fu, Yongqing Fu, Huigao Duan

**Affiliations:** ^1^ College of Mechanical and Vehicle Engineering Hunan University Changsha China; ^2^ Nursing Department Third Xiangya Hospital Central South University Changsha China; ^3^ Faculty of Engineering and Environment Northumbria University Newcastle upon Tyne UK

**Keywords:** AI, cardiac arrhythmia diagnosis, ECG, ECM, hydrogel

## Abstract

Soft bioelectronics provide a powerful platform for physiological monitoring, yet conventional hydrogel‐based devices are constrained by inherent trade‐offs between thickness, robustness, and multifunctionality, restricting their adaptability across diverse epidermal and implantable applications. Here, a novel cold‐lamination strategy is developed to mechanically interlock a TPU nanomesh within a temperature‐responsive hydrogel network, mimicking the structure of the extracellular matrix. This method enables the fabrication of devices with precise control of thickness (17–90 µm), tunable Young's modulus (36.5–761.1 kPa), excellent breathability, and large‐area scalability (> 225 cm^2^). Furthermore, the resulting bioelectronics exhibit reversible, on‐demand adhesion enabled by switchable hydrogen‐bond interactions. The ultrathin hydrogels exhibit exceptional conformability and durability, with a 17 µm film achieving tensile stress of 835.23 kPa and toughness of 1.57 MJ m^−3^, thereby overcoming the fragility of conventional ultrathin films. By forming stable tissue interfaces, they enabled reliable epidermal electrocardiogram monitoring under daily and clinical conditions and served as implantable cardiac patches for arrhythmia detection in mice. When integrated with a dual‐branch deep learning network, the intelligent platform achieved 99% in vivo classification accuracy of ventricular tachycardia, ventricular fibrillation, and other arrhythmias. Together, these results establish a broadly adaptable hydrogel system, offering a universal and scalable strategy for next‐generation bioelectronics tailored to diverse organ‐specific demands.

## Graphical abstract

A cold‐lamination strategy is introduced to fabricate ultrathin, nanomesh‐reinforced hydrogel bioelectronics with controlled thickness, tunable mechanics, and reversible adhesion. By mechanically interlocking a TPU nanomesh within a temperature‐responsive hydrogel, the platform enables robust epidermal and implantable cardiac interfaces, supporting reliable ECG monitoring and intelligent in vivo arrhythmia classification.

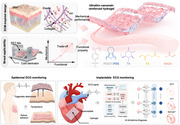

## 1 Introduction

The rapid growth of the globally aging population is driving a surge in demand for advanced remote medical and personal healthcare systems. In this context, soft bioelectronics has emerged as a powerful platform for comfortable, stable, and high‐fidelity physiological monitoring, enabling timely disease prevention, detection, diagnosis, and treatment [1, 2]. A paramount challenge for both cutaneous and implantable devices, however, is to achieve a seamless mechanical match with biological tissues, a critical requirement for ensuring high‐fidelity signal acquisition and minimizing adverse tissue reactions. Conventional electronic devices, predominantly fabricated from materials such as metals, ceramics, and hard plastics, are inherently rigid, solid, and mechanically mismatched with the soft, wet, and dynamic nature of biological structures or environments (with an elastic modulus range from ∼1 kPa to 1 MPa) [3]. This often compromises signal quality, causes user discomfort, and triggers inflammation at the tissue‐device interface. Furthermore, application scenarios impose distinct functional requirements for these devices. For example, the epidermal devices must be breathable and comfortable, whereas the implantable systems necessitate ultra‐conformability and are imperceptible to mitigate foreign‐body responses and enhance comfort [4, 5]. Hence, developing innovative strategies to simultaneously bridge this mechanical mismatch and integrate multifunctional capabilities is imperative for realizing next‐generation, high‐performance bioelectronic systems.

Over the past two decades, advances in bioelectronics have been driven by numerous strategies encompassing both novel materials and innovative structural designs. Among these, soft but solid‐like hydrogels have been extensively investigated as a promising functional material for bioelectronics due to their tissue‐like mechanical properties, high bio‐affinity, inherent ionic conductivity, and ability to maintain epidermal hydration [6, 7, 8]. Furthermore, additional functionalities critical for specific applications, such as controllable adhesion, antibacterial properties, and photoluminescence, can be engineered through rational cross‐linking design and strategic incorporation of functional components. For example, Zhang et al. developed a cellulose nanofiber–mediated supramolecular hydrogel strategy with reversible tough adhesion and facile photodetachment [9]. Generally, the hydrogel possesses a high‐water content of over 90% [10], which endows them with excellent softness. However, this weakens its mechanical strength and makes it not easy to be used as a self‐standing film [11]. Consequently, many current bioelectronic‐utilized hydrogels are often made into millimeter‐scale thickness, which creates a substantial gap between the electrode and biological tissue, impeding signal acquisition, increasing contact impedance, introducing motion artifacts, and affecting measurement accuracy [12, 13]. In contrast, ultrathin hydrogel architectures are paramount for high‐performance bioelectronics, enabling critical attributes such as excellent conformability to dynamic tissues, high permeability for long‐term wearability, and overall imperceptibility. Hence, developing an ultra‐thin hydrogel‐based platform with tunable functionality and mechanical properties represents a promising strategy for enabling bioelectronic integration across a wide range of tissues, for applications from the skin to the heart.

The challenges of simultaneously balancing thickness, mechanical performance, and multifunctionality in hydrogel bioelectronics can be addressed by drawing inspiration from the sophisticated architecture of human skin and internal organs, which provide a natural design paradigm for next‐generation ultrathin hydrogel devices. For instance, the extracellular matrix (ECM) is comprised of a stiff collagen fibril scaffold interwoven with a compliant elastin network, endowing biological tissues with an exceptional fracture resistance, elasticity, and softness. Inspired by this architecture, researchers have reinforced hydrogels with nanofibers, porous foam, and nanotubes to substantially enhance their mechanical performance while closely mimicking the properties of native tissues [14]. Accordingly, various methods, including cast‐molding, spin‐coating, and blade‐coating, have been widely explored to fabricate such elastic fiber‐reinforced, hierarchically structured hydrogels [15, 16, 17]. However, these techniques have their inherent drawbacks. For example, the conventional casting generally yields films thicker than 500 µm, resulting in limited breathability and poor conformability [18, 19]. Spin‐coating can achieve film thicknesses on the order of tens of micrometers but requires highly hydrophilic substrates and often induces strong adhesion, complicating the subsequent transfer and integration [15, 20]. Consequently, prevailing fabrication strategies tend to yield hydrogels that are either too thick and impermeable, or too thin and fragile for practical use. Crucially, these intrinsic limitations undermine the universality of hydrogels, impeding their adaptation across diverse biological interfaces ranging from epidermal sensing to implantable monitoring. Thus, establishing a robust strategy for fabricating ultrathin functional hydrogels with precisely controlled thickness and tunable mechanical properties becomes an urgent priority for advancing their broad biomedical applications.

**FIGURE 1:**
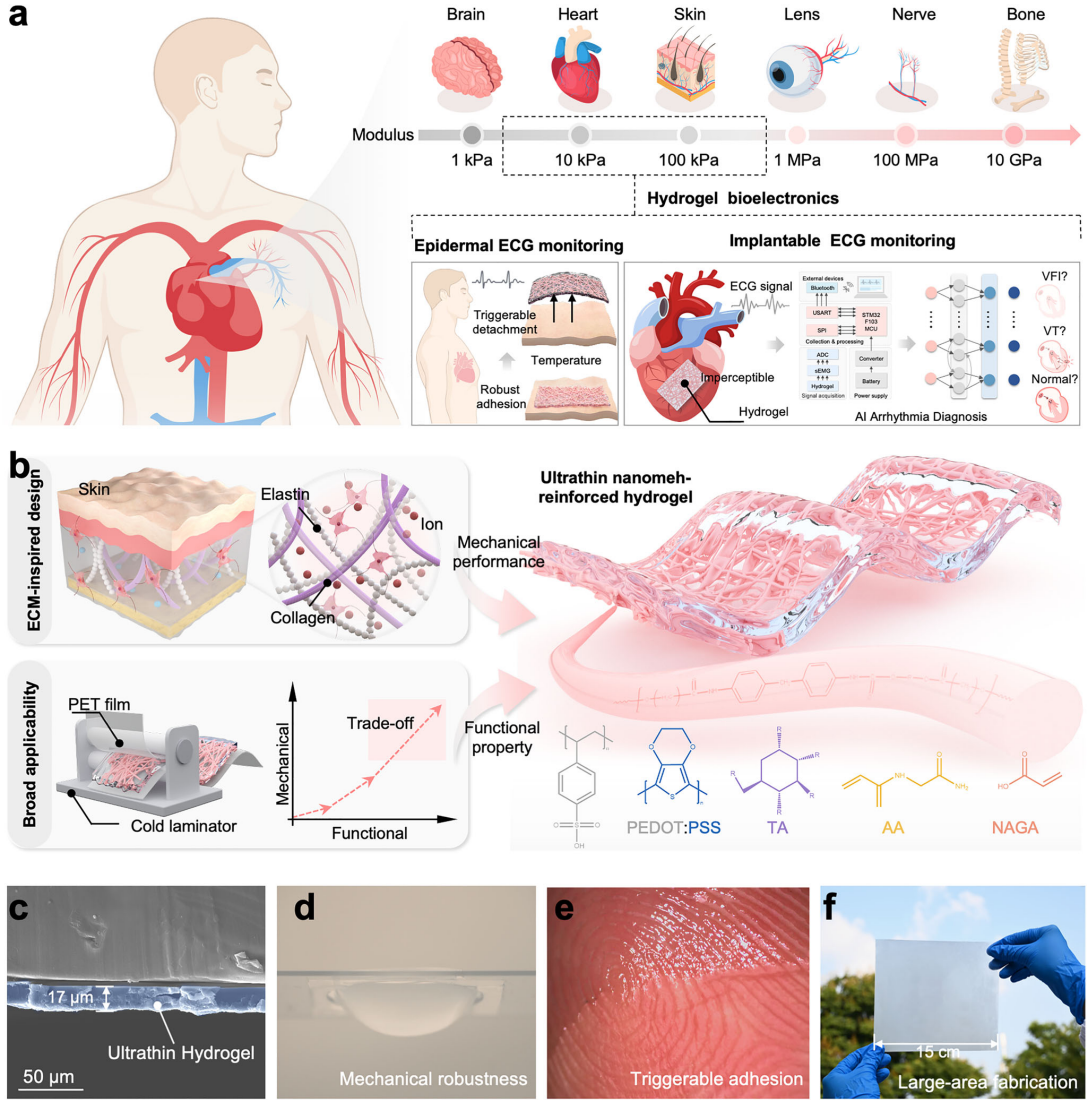
Schematic illustrations of the ultrathin nanomesh‐reinforced hydrogels. (a) Schematic illustrations of the hydrogel electronics for broad applicability. (b) Design and fabrication of ultrathin nanomesh‐reinforced hydrogels. (c) Cross‐sectional SEM image of the ultrathin hydrogel. Photographs of the ultrathin hydrogel (d) supporting water, (e) adhering to a pig heart, and (f) large‐area fabrication.

In this work, inspired by the hierarchical architecture of the ECM, we developed a cold‐lamination strategy that mechanically interlocks thermoplastic polyurethane (TPU) nano‐meshes within temperature‐responsive poly(N‐acryloylglycinamide‐co‐acrylic acid) networks. This bioinspired design circumvents the fabrication‐imposed trade‐offs of conventional approaches (where the produced hydrogels are either too thick and impermeable or too thin and fragile), and achieves thickness control in the range of 17–90 µm, delivering tunable Young's modulus from 36.5 to 761.1 kPa, with excellent breathability, and large‐area scalability (> 225 cm^2^). Figure 1 Moreover, the incorporation of reversible hydrogen‐bond interactions affords reliable on‐demand adhesion, with an on/off adhesive energy ratio of 20.7 and adhesive energy 19.6‐fold lower than commercial electrodes after ice application. These features collectively establish a broadly adaptable hydrogel platform capable of bridging mechanical mismatches across diverse tissues. For instance, we demonstrated that the 17 µm‐thick film achieved a tensile stress of 835.23 kPa and toughness of 1.57 MJ/m^3^, overcoming the fragility typically associated with ultrathin hydrogels. By balancing conformability, durability, and adhesion, our system enabled a stable epidermal electrocardiogram (ECG) monitoring during diverse daily and clinical activities, as well as implantable cardiac monitoring for five arrhythmia types in mice. Furthermore, when integrated with a dual‐branch deep learning framework, the platform achieved in vivo arrhythmia detection with 99% accuracy, underscoring its potential as the next‐generation, tissue‐adaptive bioelectronic interface  .

## 2 Design and Characterization of Ultrathin Nanomesh‐Reinforced Hydrogels

**FIGURE 2:**
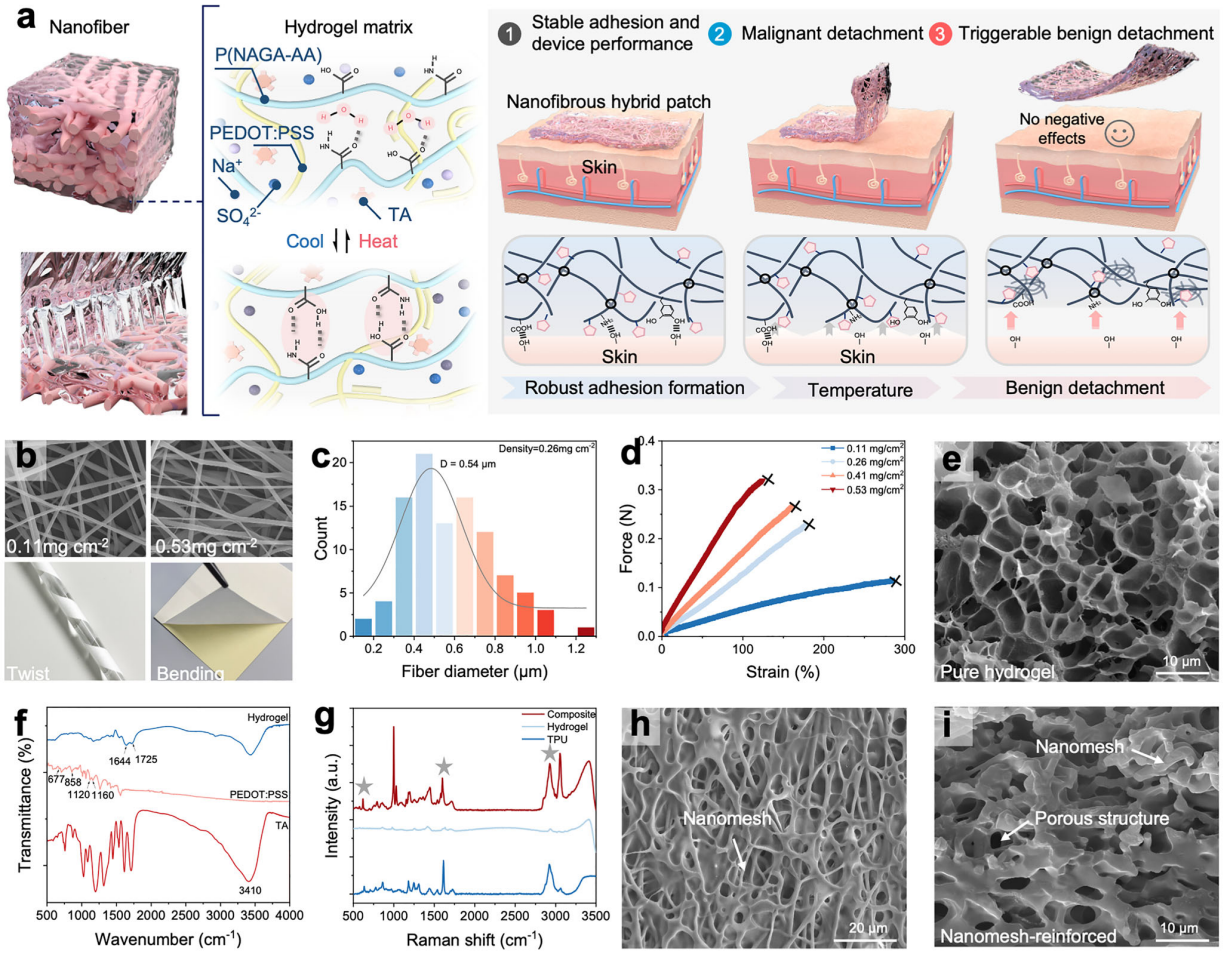
Characterization of the nanomesh‐reinforced hydrogels. (a) Material and design of thermo‐responsive nanomesh‐reinforced hydrogels. (b) SEM and optical images of TPU nanomesh scaffolds with varying densities. (c) Fiber diameter of the TPU Nanomesh scaffold. (d) Tensile curves of TPU nanomesh scaffolds. (e) SEM image of the hydrogel matrix. (f) FTIR spectrum of the hydrogel matrix. (g) Raman spectra of nanomesh, hydrogel, and ultrathin composite hydrogel. (h, i) SEM images of ultrathin nanomesh‐reinforced hydrogel.

To overcome the inherent trade‐off between mechanical robustness and ultrathin geometries in hydrogels, we followed the excellent fibrillar architectures of the natural ECM and embedded an electrospun TPU nano‐mesh scaffold into the hydrogel matrix through a cold lamination process. Figure 2a In this way, we have mimicked to form a structure with both the stiff collagen fibril scaffold and the elastic interwoven elastin matrix, similar to the ECM. To endow the hydrogels with robust adhesion and precisely controlled detachment properties, we further applied a UV‐mediated covalent crosslinking strategy. In this system, poly(N‐acryloylglycinamide‐co‐acrylic acid) (P(NAGA‐co‐AA)) networks were served as thermo‐responsive elements, in which the reversible phase separation behavior enabled dynamic modulation of hydrogen‐bonding interactions upon temperature variations. The conductive polymer poly(3,4‐ethylenedioxythiophene): poly(styrene sulfonate) (PEDOT: PSS) was incorporated to form a mechanically reinforced and electrically conductive framework within the hydrogel matrix. Tannic acid (TA) was also introduced as an adhesive component, enhancing interfacial adhesion energy, while Na_2_SO_4_ was introduced to increase the hydrogel's conductivity. The hydrogel precursor solution was cast between a PET film and a TPU nanomesh supported on a silicone‐coated release paper, and then squeezed through the gap between two rollers. The resulting hydrogel precursor film, encapsulated by the PET layer, was subsequently exposed to UV irradiation to induce its in situ gelation.

As shown in Figure 2b and Figure S1, the density of the nanomesh scaffold is increased with the increased electrospinning durations (i.e., 10, 20, 30, and 40 min), resulting in enhanced mechanical strength. The electrospun fibers exhibited an average diameter of 0.54 µm (Figure 2c), and the TPU mats demonstrated an excellent mechanical integrity, accommodating various modes of deformation. Among these samples, the nanomeshes produced with a 20‐min electrospinning duration exhibited the most favorable mechanical performance, making them the optimal scaffolds for the subsequent processing and device integration (Figure 2d). The scanning electron microscope (SEM) image of the hydrogel (Figure 2e) reveals its porous microstructure. The Fourier transform infrared spectroscopy (FTIR) of the hydrogel matrix is shown in Figure 2f. The PEDOT: PSS spectrum displays the characteristic peaks at 677 and 858 cm^−1^ (thiophene ring), as well as doublets at 993/1010 cm^−1^ (C–S stretching) and 1120/1160 cm^−1^ (C–O stretching and sulfoxide) [21]. The spectrum of tannic acid (TA) presents a broad but intense band at 3410 cm^−1^, corresponding to the O–H stretching mode and extensive hydrogen bonds. For the hydrogel, the distinct FTIR peaks can be assigned to the amino (1644 cm^−1^) and carboxyl (1725 cm^−1^) groups of NAGA and AA, together with TA‐related features clearly observed [22].

Raman spectroscopy was employed to verify the presence of TPU nanomesh scaffold within the ultrathin nanomesh‐reinforced hydrogels (Figure 2g). Top‐view SEM images further verified the successful embedding of the TPU nanomesh scaffold into the composite structure. After the freeze–drying process, the ultrathin hydrogel exhibited a three‐dimensional porous polymer network composed of randomly distributed fibers (Figure 2h). Cross‐sectional SEM images revealed that the TPU nanomesh scaffold was interlocked and embedded within the porous hydrogel matrix (Figure 2i). Uniform distribution of all the constituent elements in the ultrathin hydrogels was confirmed by performing cross‐sectional elemental mapping (Figure S2). Rheological analysis demonstrated that the storage modulus of the nanomesh‐reinforced hydrogel substantially exceeded its loss modulus, indicating its predominantly elastic behavior (Figure S3). Furthermore, thermogravimetric analysis (TGA) results quantified the hydrogel content within the TPU hybrid gel (Figure S4), which is approximately 74% of the hybrid structure.

## 3 Mechanical Properties of Ultrathin Nanomesh‐Reinforced Hydrogels

**FIGURE 3:**
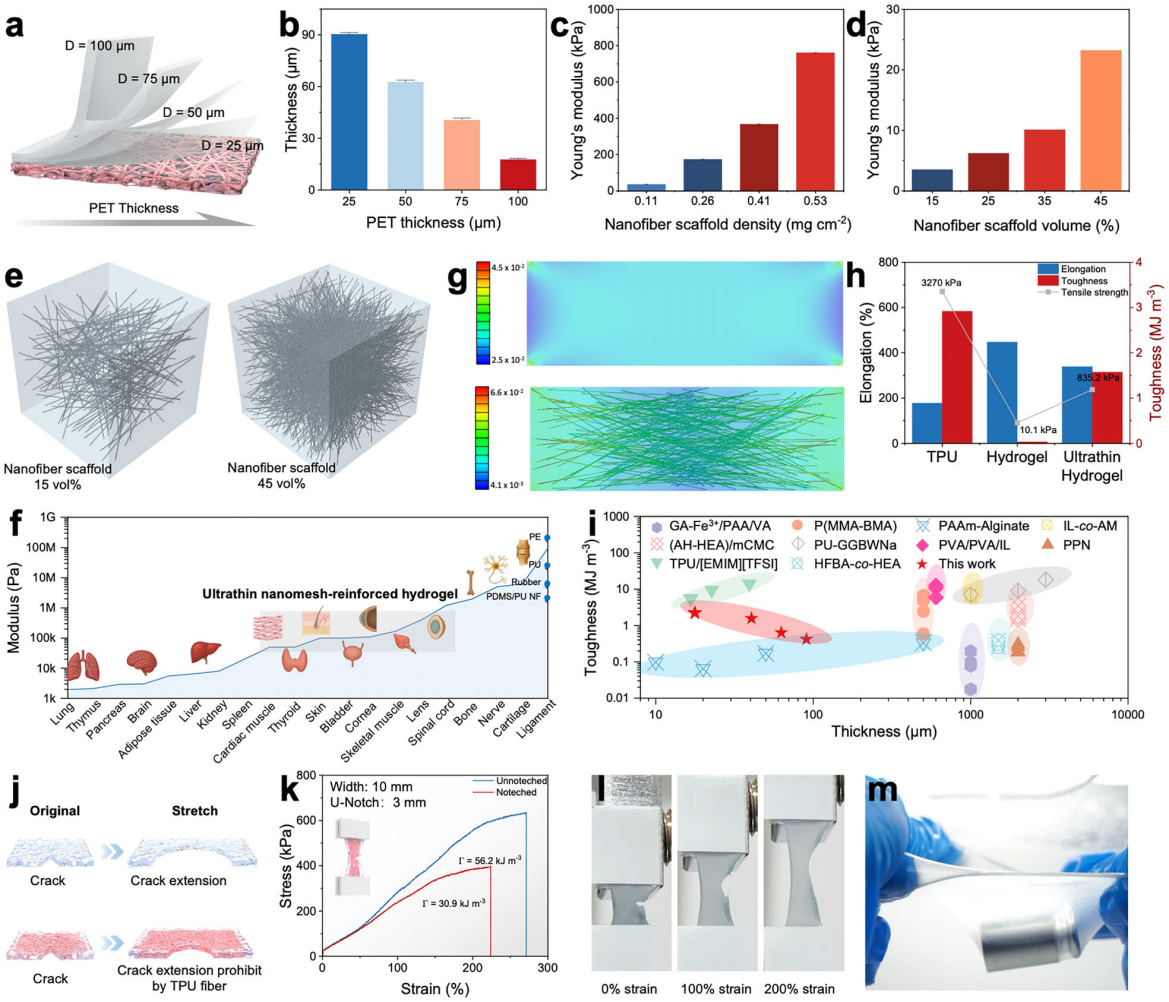
Mechanical properties of ultrathin nanomesh‐reinforced hydrogels. (a‐b) Schematic of the hydrogel showing thickness variation with different PET film thicknesses. (c) Young'smodulus with different nanomesh scaffold densities. (d) Simulated Young's modulus of different nanomesh scaffold volumes. (e) Computational model of Young's modulus. (f) Modulus matching range of our ultrathin nanomesh‐reinforced hydrogel with biological tissues and organs. (g) Stress distribution map from simulation. (h) Comparison of mechanical properties among the nanomesh scaffold, hydrogel matrix, and composite hydrogel. (i) Comparison of toughness and thickness of ultrathin nanomesh‐reinforced hydrogel with literature values. (j) Schematic illustration of the tear resistance of the ultrathin nanomesh‐reinforced hydrogel. (k) Stress–strain curves of notched and unnotched ultrathin nanomesh‐reinforced hydrogels. (l) Photographs of a notched ultrathin nanomesh‐reinforced hydrogel. (m) Photographs of the ultrathin hydrogel supporting a 50 g weight.

Unlike traditional techniques, the cold‐lamination approach we applied in this study enables precise control over film thicknesses ranging from a few to several hundred micrometers, while maintaining exceptional uniformity across large areas. By adjusting the thickness of the PET films, the obtained hydrogel film thickness can be finely tuned (Figure 3a and Table S1). As a demonstration, hydrogel films with thicknesses from 17 µm to over 90 µm were successfully produced (Figure 3b and Figure S5). The mechanical performance of these ultrathin nanomesh‐reinforced hydrogels is strongly influenced by the density of the TPU nanomesh scaffold. As shown in Figure 3c and Figure S6, increasing the scaffold density results in increased Young's modulus and improved toughness. Consistently, simulation results (Figure 3d,e) reveal the same trend, confirming the reinforcing effect of the nanomesh architecture. Furthermore, the hydrogels prepared with varying PET film thicknesses exhibited an increase in Young's modulus by nearly 3.5‐folds (i.e., from 92.32 to 324.7 kPa) (Figure S7 and Table S2). This enhancement arises from more extensive cross‐linking formed in thinner samples under identical UV exposure, yielding stiffness compatible with soft skin contact while facilitating detachment from PET substrates [23]. The tunable modulus of hydrogels, ranging from tens to several hundreds of kilopascals, allows precise mechanical matching with various biological tissues and organs [24] (Figure 3f). Such good capability, which is associated with significantly higher moduli than those previously reported (Figure S8), is difficult to achieve with the conventional thin films such as Polyurethane (PU), Polyimide (PI), Polyethylene (PE), or Polydimethylsiloxane (PDMS). Bioelectrodes fabricated from these nanomesh‐reinforced hydrogels demonstrate excellent attachment and conformability to soft tissues (Figures S9 and S10).

The ultrathin nanomesh‐reinforced hydrogel with an ECM‐like architecture can resolve the inherent conflict between achieving ultrathin geometries and maintaining sufficient mechanical strength in hydrogel systems [15, 25]. Simulation results corroborate the reinforcing roles of the electrospun nanofiber scaffold. As shown in Figure 3g, the pristine hydrogel exhibits a relatively homogeneous yet weak stress distribution, with stress concentration localized only at the corners under tensile loading. In contrast, the nanofiber‐reinforced hydrogel displays a remarkedly heterogeneous stress profile, where the embedded fibers effectively redistribute and withstand part of the applied load. This fiber‐mediated load transfer reduces local stress concentration within the hydrogel matrix and mimics the collagen fibril network in the ECM, which interweaves with elastic components to achieve both strength and compliance [14]. As demonstrated in Figure 3h and Figure S11, the ultrathin nanomesh‐reinforced hydrogel (≈17 µm) exhibits a tensile strength of 835.2 kPa, which is over 82.7‐folds higher than that of the pure hydrogel (10.1 kPa, ≈1000 µm). Its elongation at break reaches 338.7%, approximately 128% greater than that of the TPU nanomesh scaffold alone. Furthermore, the nanomesh‐reinforced hydrogel demonstrates a high toughness of 1.57 MJ·m^−^^3^, nearly 52.3 times that of the pure hydrogel (0.03 MJ·m^−^^3^). The sample maintains a linear reversible stress–strain behavior under varying loading–unloading cycles and shows its negligible dependence on the stretching rate (Figures S12 and S13). Cyclic tests were further performed to evaluate the durability of the hydrogel system. As shown in Figure S14, the first loading–unloading cycle exhibits a pronounced hysteresis loop due to viscoelastic energy dissipation, whereas the subsequent cycles display nearly overlapping stress–strain curves, indicating excellent fatigue durability arising from reversible physical interactions within the network [26]. In addition, the hydrogel–TPU interface remains intact after 1000 cycles of repeated bending, without any observable interlayer detachment, delamination, or crack formation (Figure S15). A comparative analysis of mechanical performance compared with those reported in literature (Figure 3i; Figure S16 and Table S3) further reveals that the nanomesh‐reinforced hydrogels exhibited markedly enhanced toughness, surpassing those of previously reported hydrogel‐based sensors [15, 27, 28, 29, 30, 31, 32, 33, 34, 35, 36, 37, 38, 39, 40, 41].

The nanomesh scaffold also imparts the ultrathin nanomesh‐reinforced hydrogel with exceptional puncture resistance. As demonstrated in Figure 3j, the embedded scaffold extends the force transfer length owing to its high fiber‐to‐matrix modulus ratio, thereby effectively hindering crack propagation and enhancing its tear resistance [42]. In comparison, the pure hydrogel was fractured at a strain of only 151.3% (∼33.9% of crack‐free samples; Figure 3k and Figure S17), whereas the ultrathin nanomesh‐reinforced hydrogel sustained the strains up to 223% (∼82% of crack‐free samples) even in the presence of cracks (Figure 3l). This enhanced damage tolerance further translates into superior load‐bearing and puncture resistance. Remarkably, a freely suspended ultrathin nanomesh‐reinforced hydrogel was able to support a 50 g weight without rupture (Figure 3m). Furthermore, the nanomesh scaffold enabled the hydrogel to withstand localized stresses from a screw tip and sustain loads approximately 3294 times its own weight without structural failure (Figure S18). Importantly, the cold‐laminated strategy is generalizable and applicable to other polymer systems, such as alginate‐based hydrogels (Figure S19).

## 4 Tunable Conformability and Flexibility in a Broad Range

**FIGURE 4:**
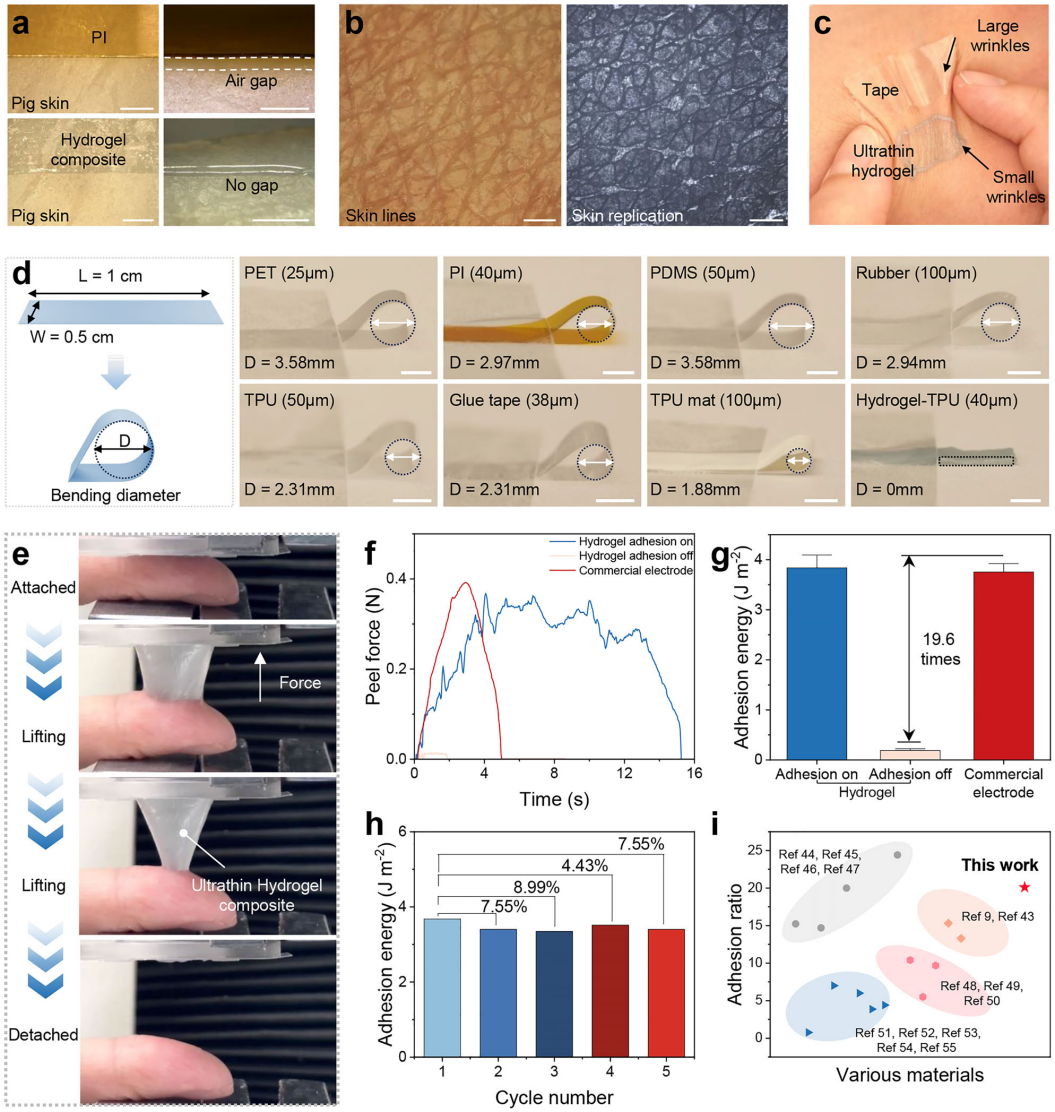
Conformability and adhesive characteristics of the ultrathin nanomesh‐reinforced hydrogel. (a) Optical images and (b)Optical microscopic image of the skin and the peeled‐off ultrathin nanomesh‐reinforced hydrogel surface from human skin (scale bar: 500 µm). (c) Wrinkles of ultrathin nanomesh‐reinforced hydrogel and PET tape under skin compression. (d) Schematic diagram and digital images showing the evaluation of material softness based on bending diameter for different materials. (e) The ultrathin nanomesh‐reinforced hydrogel separation from skin in a tack test. (f) Force–stroke curves and (g) comparison of adhesion energy between ultrathin nanomesh‐reinforced hydrogel and commercial electrode. (h) Cyclic adhesion energy of ultrathin nanomesh‐reinforced hydrogel. (i) Comparison of adhesion performance.

The conformability of a material to rough surfaces depends monotonically on its modulus and thickness. A better conformity is normally achieved through reduced modulus and thinner membranes, whereas stiffer films require smaller critical thicknesses to achieve their full attachment. Compared with those conventional stiff polymer films (modulus ∼ GPa) such as Parylene, PET, and PI, the nanomesh‐reinforced hydrogels exhibited exceptional softness, enabling seamless adhesion to rough or irregular surfaces. To experimentally validate these attributes, the skin conformability of the ultrathin nanomesh‐reinforced hydrogel was demonstrated by applying it to pig skin, with a 50 µm PET adhesive tape used for comparisons. As shown in Figure 4a, the ultrathin hydrogel established a seamless interface with the skin surface, and similar results were observed on human skin samples (Figure S20). The microfiber composite hydrogel was gently conformed to the skin's glyphic lines without forming apparent air gaps, whereas the PET tape exhibited noticeable air pockets between itself and the skin valleys due to its higher stiffness. The surface amplitude was nearly vanished in areas covered by PET tape, demonstrating the poor adaptability of rigid materials to rough surfaces. Figure 4b further illustrates the ultra‐conformal and seamless integration of the ultrathin hydrogel with the skin, as evidenced by the faithfully replicated microscopic skin texture on the peeled‐off hydrogel surface. Moreover, these hydrogels effectively accommodated various dynamic deformations on the human skin, including squeezing, stretching, and bending (Figure S21). When the skin‐mounted nanomesh‐reinforced hydrogel was squeezed, it formed fine wrinkles while maintaining intimate contact with the skin, whereas the rigid PET adhesive tape exhibited limited flexibility due to its modulus mismatch with the skin (Figure 4c and Figure S22).

To evaluate the ultrasoft characteristics of the nanomesh‐reinforced hydrogels, their bending behavior was characterized by measuring their maximum bending diameters, and the obtained results are shown in Figure 4d. Due to their exceptional softness, these hydrogels exhibited nearly flat conformations with negligible bending angles (∼0°). In contrast, the conventional flexible electronic films typically formed apparent arcs during bending, with radii around 2 mm, indicative of their relatively high stiffness. As illustrated in Figure S23, the nanomesh‐reinforced hydrogels consistently exhibited minimal bending angles with varied thicknesses of hydrogels, highlighting their remarkable mechanical compliance.

Beyond conformability, the nanomesh‐reinforced hydrogels also demonstrated excellent adhesion on various substrate surfaces with a wide range of roughness values, including objects with millimeter‐scale amplitude variations that conventional plastic films cannot easily conform to (Figure S24). To evaluate the adhesive characteristics of the ultrathin nanomesh‐reinforced hydrogel, adhesion tests were conducted using a universal tensile tester. The suspended ultrathin hydrogel was gradually lifted from the substrate while the adhesion force was continuously recorded. The representative optical images capturing the delamination process between the hydrogel and human skin are presented in Figure 4e. The ultrathin nanomesh‐reinforced hydrogel exhibited on‐demand and programmable adhesion and detachment behavior (Note S1 and Figure S25, S26). Upon cooling with an ice pack, the interfacial adhesion energy was found to dramatically decrease from 3.83 to 0.19 J/m^2^, a 19.6‐fold reduction compared to that of the commercial gel electrodes (Figure 4f). Moreover, the areal adhesion energy of the ultrathin nanomesh‐reinforced hydrogel was approximately 2% higher than commercial gels, despite being 25 times thinner (Figure 4g). Therefore, the detachment of the ultrathin nanomesh‐reinforced hydrogel will not cause potential skin irritation. In particular, the hydrogels also maintained a good cyclic adhesion, with area adhesion energy exceeding 90% after repeated attach–detach cycles (Figure 4h). Overall, the ultrathin nanomesh‐reinforced hydrogel exhibited exceptional switchable adhesion between sticky and slippery states, with performance significantly surpassing that of previously reported hydrogels (Figure 4i) [9, 43, 44, 45, 46, 47, 48, 49, 50, 51, 52, 53, 54, 55].

## 5 Electrophysiological Signal Capturing Performance

**FIGURE 5:**
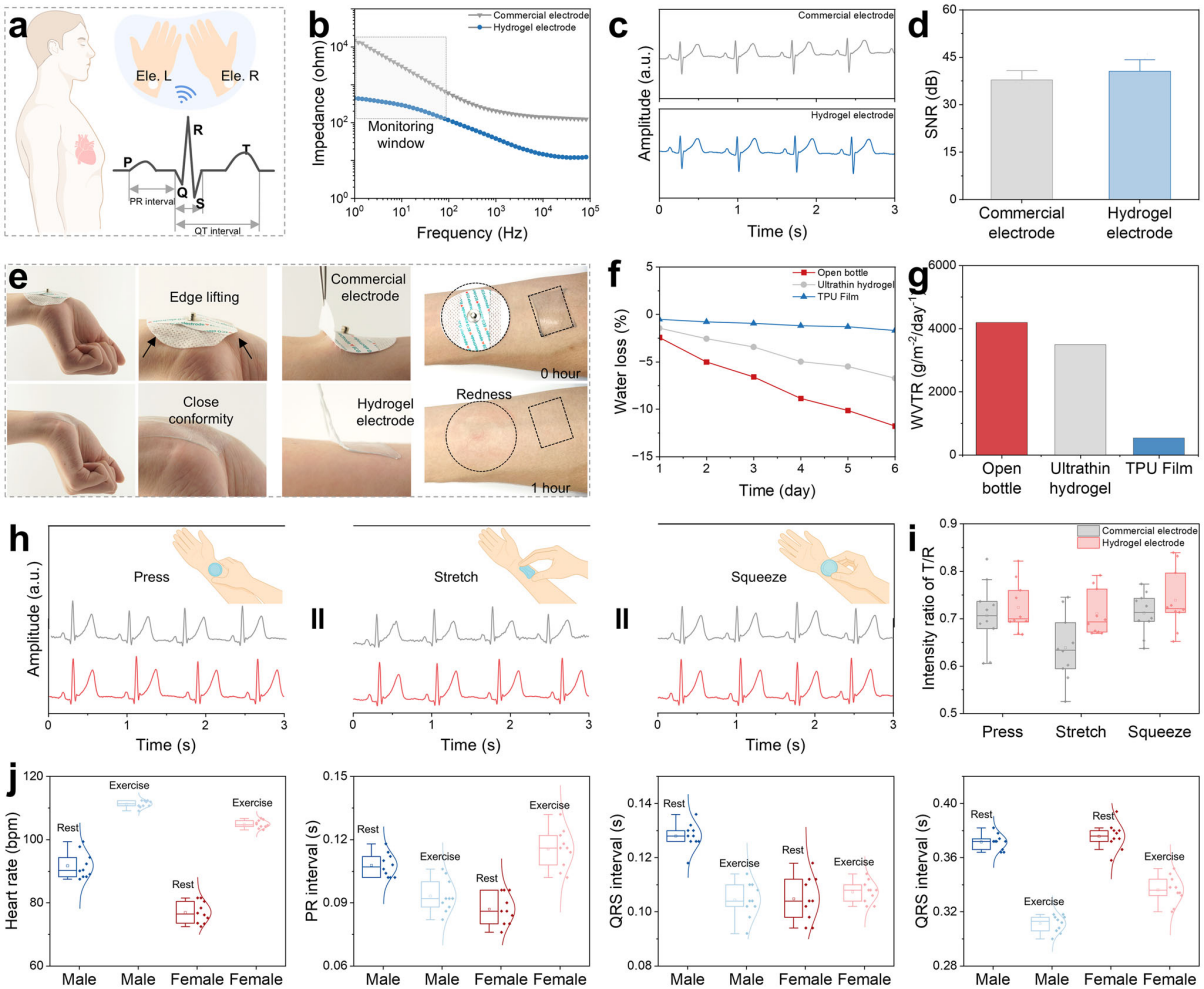
ECG data collection and analysis for personalized health care. (a) Electrode attachment positions for ECG. (b) Frequency‐dependent impedance characteristics of hydrogel electrodes and commercial electrodes. Comparison of (c) ECG signals and (d) SNR values obtained using commercial and hydrogel electrodes. (e) Comparison of conformability, adhesion, and biocompatibility. (f) Comparison of WVTR. (g) Comparison of water loss measurements. (h) ECG signals under different skin deformations, and (i) corresponding sensitivities. (j) Statistical results of heart rate, PR interval, QRS complex, and QT interval from ECG signals of different genders.

To evaluate the suitability of ultrathin nanomesh‐reinforced hydrogels for personalized health monitoring, we conducted a comparative study against the commercial Ag/AgCl gel electrodes. The hydrogel electrodes exhibited multiple advantages, including ultrathin and lightweight construction, high signal quality and stability, excellent skin conformity, and enhanced biocompatibility. Electrocardiogram (ECG) measurements were performed using a standard three‐electrode configuration, with two electrodes served as differential input terminals and a third as the reference to minimize external interference (Figure 5a). The hydrogel electrodes demonstrated significantly lower skin–electrode impedance than the commercial electrodes (Figure 5b). The recorded ECG signals displayed clear and distinct PQRST waveforms, with key features such as heart rate closely matching those obtained from the commercial electrodes (Figure 5c). Furthermore, the hydrogel electrodes achieved a higher signal‐to‐noise ratio (SNR) of 40.6 ± 3.6, compared to that of 37.9 ± 2.9 for Ag/AgCl gels (Figure 5d).

To further compare the developed ultrathin hydrogel electrodes with the commercial counterparts, we evaluated their conformability, adhesion, biocompatibility, and permeability (Figure 5e). When applied to high‐curvature skin regions, such as wrist folds, the commercial electrodes were found to peel off at the edges, whereas the ultrathin hydrogels were closely adhered and fully conformed to skin textures. Regarding its performance for detachment, commercial electrodes exhibited excessive adhesion, making removal difficult and uncomfortable. In contrast, the ultrathin hydrogels enabled painless and non‐traumatic removal by simply applying cooling (e.g., applying the ice). Biocompatibility tests demonstrated that the ultrathin electrodes could be worn for 1 h without causing apparent itchiness or inflammation, whereas commercial electrodes induced noticeable redness and swelling. Consistently, further in vitro cytotoxicity experiments showed no significant reduction in cell viability (Figure S27). Permeability tests indicated that the water mass loss of the ultrathin hydrogel over six days was slightly lower than that of the open‐bottle group but still above the reported human sweat evaporation rate (∼600 g m^−^^2^ day^−1^), suggesting its sufficient gas permeability to allow skin ventilation (Figure 5f,g).

The ultrathin hydrogels were also maintained with stable signal outputs during various skin deformations, including squeezing, pressing, and stretching (Figure 5h). Across all deformation conditions, they consistently demonstrated higher sensitivity values than the commercial gel electrodes, as quantified by the T/R wave intensity ratio (Figure 5i). We further assessed hydrogel performance across male and female volunteers under both resting and exercising conditions (Figure 5j). As expected, the heart rate was increased during exercise in both groups, and critical ECG intervals, including PR, QRS, and QT, were reliably captured under both physiological states.

## 6 Application of AI‐Enabled In Vivo ECG Arrhythmia Diagnosis

**FIGURE 6:**
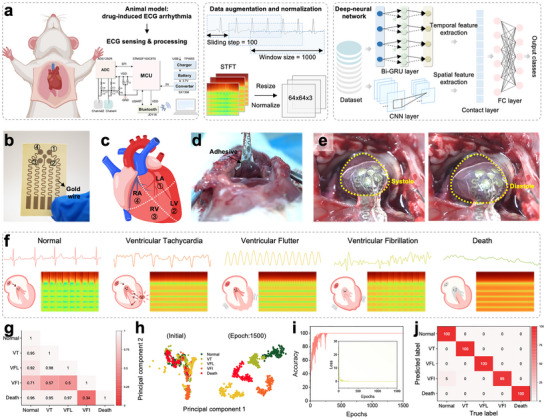
Application of AI‐enabled in vivo ECG arrhythmia diagnosis. (a, b) Schematic representation of four cardiac sensor channels on a heart model. Photographs of the cardiac patch (c) adhered to the surface of a rat's heart and (d) conforming to cardiac contraction and relaxation. (e) ECG signals with corresponding STFT of five cardiac events. (f) Overview of the intelligent cardiac arrhythmia diagnosis system. (g) Correlation matrix of the ECG signals for the five cardiac events. (h) t‐SNE of ECG signals before and after neural network training. (i) Loss and accuracy curves over epochs. (j) Confusion matrix for arrhythmia classification.

We further monitored various in vivo ECG arrhythmias in animal models using the ultrathin nanomesh‐reinforced hydrogels as a biointerface cardiac patch coupled with a signal acquisition board and deep learning algorithms (Figure 6a). The arrhythmias were induced by administration of aconitine, which inhibits the inactivation of sodium channels in cardiomyocytes, thereby keeping them persistently open and leading to sustained depolarization and abnormal heart rhythms, including normal rhythm, ventricular tachycardia (VT), ventricular flutter (VFL), ventricular fibrillation (VFI), and cardiac arrest (death). A flexible ultrathin cardiac patch was developed with four channels connected to the left ventricle and right atrium (Figure 6b,c). The patch exhibited a strong adhesion to the heart and was conformed well to cardiac contractions and relaxations (Figure 6d,e). From Figure 6f, the resulting ECG signals provided a clear and distinct visualization of different cardiac events. For example, in the VFL, the P‐QRS‐T waves disappeared and were replaced by continuous, rapid, and relatively regular flutter waves. Whereas in the VF, no recognizable QRS complexes were observed, but instead irregular high‐frequency fibrillation waves with variable morphology, amplitude, and interval were present. Short‐time Fourier transform (STFT) was further applied to convert the time‐domain signals into a time–frequency representation, producing spectrograms with unique patterns that reflected the distinct characteristics of each arrhythmia.

To enable arrhythmia classification, a data augmentation procedure was implemented to expand the dataset of ECG signal images. Specifically, the time‐domain ECG signals were segmented into fragments using a sliding window, and STFT was applied to generate additional spectrograms for deep learning training. We adopted a dual‐branch parallel deep neural network, where a convolutional neural network (CNN) was applied to capture image‐based features and a bidirectional gated recurrent unit (Bi‐GRU) was applied to capture the temporal features. All the hyperparameter settings are summarized in Table S4. This architecture effectively fused one‐dimensional time‐series signals with two‐dimensional image representations, thereby leveraging multimodal information. As shown in Figure 6g, the correlation matrix of five arrhythmia types revealed strong interclass correlations, which may increase the risk of misclassification. To further visualize feature evolution, the t‐distributed stochastic neighbor embedding (t‐SNE) was applied (Figure 6h). Initially, substantial overlap was observed among classes. However, after 1500 training epochs, the t‐SNE visualization demonstrated well‐separated clusters with clear boundaries and minimal overlap. A small degree of confusion was observed for the VFI class, where 5% of the samples were misclassified as Normal. Overall, the developed model achieved a prediction accuracy of 99% (Figure 6i,j), indicating a high discriminative capability across all arrhythmia types.

## 7 Conclusion

In summary, we developed a bioinspired cold‐lamination strategy to engineer ultrathin hydrogel bioelectronics that simultaneously achieve precise thickness control, tunable mechanical robustness, large‐area scalability, and reversible adhesion. By mechanically interlocking TPU nanomeshes within temperature‐responsive hydrogel networks, our approach effectively overcomes the trade‐offs of conventional fabrication techniques, enabling the creation of robust yet highly conformable films that bridge the mechanical mismatch between rigid devices and soft biological tissues. The resulting platform demonstrates broad adaptability across physiological contexts, from stable, high‐fidelity epidermal ECG monitoring during daily activities to implantable cardiac patches for arrhythmia detection in vivo. Furthermore, integration with a dual‐branch deep learning framework enabled arrhythmia classification with 99% accuracy, underscoring the potential of coupling soft biointerfaces with intelligent analytics.

Despite the promising results demonstrated in this study, the current cardiac patch was implemented in a wired configuration, which may limit the portability and long‐term practicality of the system for fully wearable or ambulatory applications. Looking forward, future research will focus on developing a more highly integrated and multifunctional cardiac patch, advancing toward a diagnosis–therapy–integrated platform. Potential directions include the incorporation of on‐patch electronics and wireless communication modules to enable untethered operation, as well as the integration of therapeutic functionalities, such as controlled drug delivery or electrical stimulation, in combination with real‐time cardiac monitoring.

## 8 Methods

The detailed experimental section of the hydrogels is provided in the Supporting Information.

## Conflicts of Interest

The authors declare no conflicts of interest.

## Supporting information




**Supporting File**: advs74308‐sup‐0001‐SuppMat.docx.

## Data Availability

The data that support the findings of this study are available from the corresponding author upon reasonable request.

## References

[1] C. Zhao , J. Park , S. E. Root , and Z. Bao , “Skin‐inspired Soft Bioelectronic Materials, Devices and Systems,” Nature Reviews Bioengineering 2 (2024): 671–690, 10.1038/s44222-024-00194-1.

[2] R. Liu , Y. Liu , Y. Cheng , et al., “Aloe Inspired Special Structure Hydrogel Pressure Sensor for Real‐Time Human‐Computer Interaction and Muscle Rehabilitation System,” Advanced Functional Materials 33, no. 50 (2023): 2308175, 10.1002/adfm.202308175.

[3] H. Yuk , J. Wu , and X. Zhao , “Hydrogel Interfaces For merging Humans and Machines,” Nature Reviews Materials 7, no. 12 (2022): 935, 10.1038/s41578-022-00483-4.

[4] M. Lin , H. Hu , S. Zhou , and S. Xu , “Soft Wearable Devices for Deep‐Tissue Sensing,” Nature Reviews Materials 7, no. 11 (2022): 850, 10.1038/s41578-022-00427-y.

[5] H. Liu , D. Li , H. Chu , et al., “Ultra‐stretchable Triboelectric Touch Pad with Sandpaper Micro‐surfaces for Transformer‐assisted Gesture Recognition,” Nano Energy 130 (2024): 110110, 10.1016/j.nanoen.2024.110110.

[6] X. Li and J. P. Gong , “Design Principles for Strong and Tough Hydrogels,” Nature Reviews Materials 9, no. 6 (2024): 380, 10.1038/s41578-024-00672-3.

[7] H. C. Ates , P. Q. Nguyen , L. Gonzalez‐Macia , et al., “End‐to‐end Design of Wearable Sensors,” Nature Reviews Materials 7, no. 11 (2022): 887, 10.1038/s41578-022-00460-x. 35910814 PMC9306444

[8] H. Liu , Y. Li , Q. Sun , et al., “Triboelectric Wearable Devices for Accelerated Wound Healing,” Chemical Engineering Journal 497 (2024): 154628, 10.1016/j.cej.2024.154628.

[9] L. Zhang , L. Chen , S. Wang , et al., “Cellulose Nanofiber‐mediated Manifold Dynamic Synergy Enabling Adhesive and Photo‐detachable Hydrogel for Self‐powered E‐skin,” Nature Communications 15, no. 1 (2024): 3859, 10.1038/s41467-024-47986-y. PMC11078967 38719821

[10] C. Wang , H. Wang , B. Wang , et al., “On‐skin Paintable Biogel for Long‐term High‐fidelity Electroencephalogram Recording,” Science Advances 8, no. 20: abo1396, 10.1126/sciadv.abo1396. PMC9122322 35594357

[11] G. Ge , Y. Lu , X. Qu , et al., “Muscle‐Inspired Self‐Healing Hydrogels for Strain and Temperature Sensor,” ACS Nano 14, no. 1 (2020): 218, 10.1021/acsnano.9b07874. 31808670

[12] Y. Ohm , C. Pan , M. J. Ford , X. Huang , J. Liao , and C. Majidi , “An Electrically Conductive Silver–polyacrylamide–alginate Hydrogel Composite for Soft Electronics,” Nature Electronics 4 , no. 3 (2021): 185, 10.1038/s41928-021-00545-5.

[13] X. Xia , Q. Liang , X. Sun , et al., “Intrinsically Electron Conductive, Antibacterial, and Anti‐swelling Hydrogels as Implantable Sensors for Bioelectronics,” Advanced Functional Materials 32, no. 48 (2022): 2208024, 10.1002/adfm.202208024.

[14] J. Wang , B. Wu , P. Wei , S. Sun , and P. Wu , “Fatigue‐free Artificial Ionic Skin Toughened by Self‐healable Elastic Nanomesh,” Nature Communications 13, no. 1 (2022): 4411, 10.1038/s41467-022-32140-3. PMC9338060 35906238

[15] Q. Gao , F. Sun , Y. Li , et al., “Biological Tissue‐Inspired Ultrasoft, Ultrathin, and Mechanically Enhanced Microfiber Composite Hydrogel for Flexible Bioelectronics,” Nano‐Micro Letters 15 (2023): 139, 10.1007/s40820-023-01096-4. 37245163 PMC10225432

[16] Z. Zhang , J. Yang , H. Wang , et al., “A 10‐Micrometer‐thick Nanomesh‐reinforced Gas‐permeable Hydrogel Skin Sensor for Long‐term Electrophysiological Monitoring,” Science Advances 10, no. 2: adj5389, 10.1126/sciadv.adj5389. PMC10781413 38198560

[17] C. Zhu , G. Chen , S. Li , et al., “Breathable Ultrathin Film Sensors Based on Nanomesh Reinforced Anti‐Dehydrating Organohydrogels for Motion Monitoring,” Advanced Functional Materials 34 (2024): 2411725, 10.1002/adfm.202411725.

[18] Y. Gao , X. Han , J. Chen , et al., “Hydrogel–mesh Composite for Wound Closure,” Proceedings of the National Academy of Sciences 118, no. 28 (2021): 2103457118, 10.1073/pnas.2103457118. PMC8285977 34264848

[19] H. Lei , L. Dong , Y. Li , et al., “Stretchable Hydrogels with Low Hysteresis and Anti‐fatigue Fracture based on Polyprotein Cross‐linkers,” Nature Communications 11, no. 1 (2020): 4032, 10.1038/s41467-020-17877-z. PMC7423981 32788575

[20] Z. Wu , H. Ding , K. Tao , et al., “Ultrasensitive, Stretchable, and Fast‐Response Temperature Sensors Based on Hydrogel Films for Wearable Applications,” ACS Applied Materials & Interfaces 13, no. 18 (2021): 21854, 10.1021/acsami.1c05291. 33908749

[21] L. Sordini , J. C. Silva , F. F. F. Garrudo , et al., “PEDOT:PSS‐Coated Polybenzimidazole Electroconductive Nanofibers for Biomedical Applications,” Polymers 13, no. 16 (2021): 2786, 10.3390/polym13162786. 34451324 PMC8401200

[22] M. A. Pantoja‐Castro , “González‐Rodríguez, Study by Infrared Spectroscopy and Thermogravimetric Analysis of Tannins and Tannic Acid,” Revista Latinoamericana De Química 39, no. 3 (2011): 107.

[23] S. Cheng , Z. Lou , L. Zhang , et al., “Ultrathin Hydrogel Films Toward Breathable Skin‐Integrated Electronics,” Advanced Materials 35, no. 1 (2023): 2206793, 10.1002/adma.202206793. 36267034

[24] C. F. Guimarães , L. Gasperini , A. P. Marques , and R. L. Reis , “The Stiffness of Living Tissues and its Implications for Tissue Engineering,” Nature Reviews Materials 5, no. 5 (2020): 351, 10.1038/s41578-019-0169-1.

[25] Y. Wang , S. Lee , H. Wang , et al., “Robust, Self‐adhesive, Reinforced Polymeric Nanofilms enabling Gas‐permeable Dry Electrodes for Long‐term Application,” Proceedings of the National Academy of Sciences 118, no. 38 (2021): 2111904118, 10.1073/pnas.2111904118. PMC8463786 34518214

[26] J. Xu , G. Wang , Y. Wu , X. Ren , and G. Gao , “Ultrastretchable Wearable Strain and Pressure Sensors Based on Adhesive, Tough, and Self‐healing Hydrogels for Human Motion Monitoring,” ACS Applied Materials & Interfaces 11, no. 28 (2019): 25613, 10.1021/acsami.9b08369. 31273992

[27] Z. Zhang , J. Yang , H. Wang , et al., “A 10‐Micrometer‐thick Nanomesh‐reinforced Gas‐permeable Hydrogel Skin Sensor for Long‐term Electrophysiological Monitoring,” Science Advances 10, no. 2 (2024): adj5389, https://doi.org, 10.1126/sciadv.adj5389. PMC10781413 38198560

[28] Y. Wu , J. Qu , X. Zhang , et al., “Biomechanical Energy Harvesters Based on Ionic Conductive Organohydrogels via the Hofmeister Effect and Electrostatic Interaction,” ACS Nano 15, no. 8 (2021): 13427, 10.1021/acsnano.1c03830. 34355557

[29] J. Zhang , Z. Ma , M. Li , M. Lou , H. Wang , and L. Jia , “Development of Ultrathin, Breathable, Waterproof, and Durable Nanonet‐Supported Ionogel Sensors for Electrophysiological Monitoring,” Advanced Functional Materials 35, no. 8 (2025): 2415694, 10.1002/adfm.202415694.

[30] L. You , X. Shi , J. Cheng , et al., “Flexible Porous Gelatin/Polypyrrole/Reduction Graphene Oxide Organohydrogel for Wearable Electronics,” Journal of Colloid and Interface Science 625 (2022): 197–209, 10.1016/j.jcis.2022.06.041. 35716615

[31] X. Liu , Z. Huang , C. Ye , et al., “Graphene‐Based Hydrogel Strain Sensors With Excellent Breathability for Motion Detection and Communication,” Macromolecular Materials and Engineering 307, no. 8 (2022): 2200001, 10.1002/mame.202200001.

[32] H. Xue , D. Wang , M. Jin , et al., “Hydrogel Electrodes with Conductive and Substrate‐adhesive Layers for Noninvasive Long‐term EEG Acquisition,” Microsystems & Nanoengineering 9, no. 1 (2023): 79, 10.1038/s41378-023-00524-0. 37313471 PMC10258200

[33] X.‐F. Zhang , X. Ma , T. Hou , et al., “Inorganic Salts Induce Thermally Reversible and Anti‐Freezing Cellulose Hydrogels,” Angewandte Chemie International Edition 58, no. 22 (2019): 7366, 10.1002/anie.201902578. 30938928

[34] S. Yan , Y. Chen , D. Li , et al., “Mechanically Robust, Transparent, Conductive Hydrogels based on Hydrogen Bonding, Ionic Coordination Interactions and Electrostatic Interactions for Light‐curing 3D Printing,” Chemical Engineering Journal 486 (2024): 150289, 10.1016/j.cej.2024.150289.

[35] S. Xiang , X. He , F. Zheng , and Q. Lu , “Multifunctional Flexible Sensors based on Ionogel Composed Entirely of Ionic Liquid with Long Alkyl Chains for Enhancing Mechanical Properties,” Chemical Engineering Journal 439 (2022): 135644, 10.1016/j.cej.2022.135644.

[36] T. Ye , X. Zhang , J. Wen , X. Sun , D. He , and W. Li , “Multifunctional Visualized Electronic Skin based on a Solvatochromic poly (ionic liquid) Ionogel,” Chemical Engineering Journal 477 (2023): 147182, 10.1016/j.cej.2023.147182.

[37] M. Pi , S. Qin , S. Wen , et al., “Rapid Gelation of Tough and Anti‐Swelling Hydrogels Under Mild Conditions for Underwater Communication,” Advanced Functional Materials 33, no. 1 (2023): 2210188, 10.1002/adfm.202210188.

[38] H. Peng , F. Yang , X. Wang , et al., “Rapid Radiation Synthesis of a Flexible, Self‐Healing, and Adhesive Ionogel With Environmental Tolerance for Multifunctional Strain Sensors,” ACS Applied Materials & Interfaces 15, no. 44 (2023): 51763, 10.1021/acsami.3c12082. 37874752

[39] Y. Hao , Q. Yan , H. Liu , et al., “A Stretchable, Breathable, And Self‐Adhesive Electronic Skin With Multimodal Sensing Capabilities for Human‐Centered Healthcare,” Advanced Functional Materials 33, no. 44 (2023): 2303881, 10.1002/adfm.202303881.

[40] J. Zhou , Y. Liu , F. Zhuo , et al., “Superior Compressive and Tensile bi‐directional Strain Sensing Capabilities Achieved using Liquid Metal Hybrid‐Hydrogels Empowered by Machine Learning Algorithms,” Chemical Engineering Journal 479 (2024): 147790, 10.1016/j.cej.2023.147790.

[41] C. Wang , F. Wang , J. Liu , W. Yi , Q. Zhao , and Y. Liu , “Transdermal Drug‐delivery Motion‐sensing Hydrogels for Movement Recovery caused by External Injury,” Chemical Engineering Journal 488 (2024): 150998, 10.1016/j.cej.2024.150998.

[42] C. Zhao , Y. Wang , G. Tang , et al., “Ionic Flexible Sensors: Mechanisms, Materials, Structures, and Applications,” Advanced Functional Materials 32 (2022): 2110417, 10.1002/adfm.202110417.

[43] X. Wan , L. Jia , X. Liu , B. Dai , L. Jiang , and S. Wang , “WET‐Induced Layered Organohydrogel as Bioinspired “Sticky−Slippy Skin” for Robust Underwater Oil‐Repellency,” Advanced Materials 34, no. 16 (2022): 2110408, 10.1002/adma.202110408. 35180331

[44] N. Kim , H. Kang , J.‐H. Lee , S. Kee , S. H. Lee , and K. Lee , “Highly Conductive All‐Plastic Electrodes Fabricated Using a Novel Chemically Controlled Transfer‐Printing Method,” Advanced Materials 27, no. 14 (2015): 2317, 10.1002/adma.201500078. 25708658

[45] Y. Gao , K. Wu , and Z. Suo , “Photodetachable Adhesion,” Advanced Materials 31, no. 6 (2019): 1806948, 10.1002/adma.201806948. 30549118

[46] M. Gao , H. Wu , R. Plamthottam , et al., “Skin Temperature‐triggered, Debonding‐on‐demand Sticker for a Self‐powered Mechanosensitive Communication System,” Matter 4 (2021): 1962–1974, 10.1016/j.matt.2021.03.003.

[47] L. Zhang , S. Wang , Z. Wang , et al., “Temperature‐Mediated Phase Separation Enables Strong yet Reversible Mechanical and Adhesive Hydrogels,” ACS Nano 17, no. 14 (2023): 13948, 10.1021/acsnano.3c03910. 37428219

[48] Y. Zhang , Y. Dai , F. Xia , and X. Zhang , “Gelatin/polyacrylamide Ionic Conductive Hydrogel with Skin Temperature‐triggered Adhesion for Human Motion Sensing and Body Heat Harvesting,” Nano Energy 104 (2022): 107977, 10.1016/j.nanoen.2022.107977.

[49] Y. Zheng , M. Wu , M. Duan , et al., “Skin Temperature‐Triggered Switchable Adhesive Coatings for Wearing Comfortable Epidermal Electronics,” Chemical Engineering Journal 488 (2024): 150459, 10.1016/j.cej.2024.150459.

[50] L. Zhang , S. Wang , Z. Wang , et al., “A Sweat‐pH‐enabled Strongly Adhesive Hydrogel for Self‐powered E‐skin Applications,” Materials Horizons 10, no. 6 (2023): 2271, 10.1039/D3MH00174A. 37022102

[51] Q. Zeng , F. Wang , R. Hu , et al., “Debonding‐On‐Demand Polymeric Wound Patches for Minimal Adhesion and Clinical Communication,” Advanced Science 9, no. 29 (2022): 2202635, 10.1002/advs.202202635. 35988152 PMC9561782

[52] Y. Miao , M. Xu , and L. Zhang , “Electrochemistry‐Induced Improvements of Mechanical Strength, Self‐Healing, and Interfacial Adhesion of Hydrogels,” Advanced Materials 33, no. 40 (2021): 2102308, 10.1002/adma.202102308. 34418178

[53] L. Zhou , C. Dai , L. Fan , et al., “Injectable Self‐Healing Natural Biopolymer‐Based Hydrogel Adhesive with Thermoresponsive Reversible Adhesion for Minimally Invasive Surgery,” Advanced Functional Materials 31, no. 14 (2021): 2007457, 10.1002/adfm.202007457.

[54] L. Xue , A. Kovalev , K. Dening , et al., “Reversible Adhesion Switching of Porous Fibrillar Adhesive Pads by Humidity,” Nano Letters 13, no. 11 (2013): 5541, 10.1021/nl403144w. 24171547

[55] K. R. Jinkins , S. Li , H. Arafa , et al., “Thermally Switchable, Crystallizable Oil and Silicone Composite Adhesives for Skin‐interfaced Wearable Devices,” Science Advances 8, no. 23 (2022): abo0537, 10.1126/sciadv.abo0537. PMC9187235 35687686

