# Supplementary material for: Bioinspired Cold‐Laminated Ultrathin Hydrogels as a Broadly Adaptive Platform for Physiological Monitoring
Source: Adv Sci (Weinh). 2026 Feb 15;13(22):e24317. doi: 10.1002/advs.202524317 (PMC13088340; doi:10.1002/advs.202524317)
Supplement: Supplementary file 1 — Supporting File: advs74308‐sup‐0001‐SuppMat.docx. [file ADVS-13-e24317-s001.docx]

**Supporting information for**

**Bioinspired Cold-Laminated Ultrathin Hydrogels as a Broadly Adaptive Platform for Physiological Monitoring**

**Authors**

*Hui Chen^1^, Jian Zhou^1,*^, Jianfei Xie^2,*^ , Lin Shi^1^, Lu Wang ^1^, Yuanfan Yang ^1^, Yihao Guo^1^, Jianhui Cao^1^, Qingyun Fu^1^, Yongqing Fu^3^ , Huigao Duan^1,*^*

**Affiliations**

1. College of Mechanical and Vehicle Engineering, Hunan University, Changsha 410082, China

2. Nursing Department, Third Xiangya Hospital, Central South University, Changsha 410013, China

3. Faculty of Engineering and Environment, Northumbria University, Newcastle upon Tyne, NE1 8ST, United Kingdom

* Corresponding E-mail address: [jianzhou@hnu.edu.cn](http://jianzhou@hnu.edu.cn), [xiejianfei@csu.edu.cn](http://xiejianfei@csu.edu.cn), [duanhg@hnu.edu.cn](http://duanhg@hnu.edu.cn)

# Supporting display items:

- **Supplementary Methods**
- **Supplementary Table S1.** Composition of the nanomesh-reinforced hydrogel.
- **Supplementary Table S2.** Young’s moduli and tensile strain of ultrathin nanomesh-reinforced hydrogel with different thicknesses.
- **Supplementary Table S3.** Performance comparison between ultrathin nanomesh-reinforced hydrogel and reported flexible electronic skin sensors.
- **Supplementary Table S4.** Training configuration and hyperparameters of the dual-branch parallel deep neural network.
- **Figure S1.** Effect of electrospinning duration on nanofiber mat density.
- **Figure S2.** Cross-sectional mapping of the ultrathin nanomesh-reinforced hydrogel.
- **Figure S3.** Rheological properties of the ultrathin nanomesh-reinforced hydrogel.
- **Figure S4.** TGA curves of TPU nanomesh scaffold and ultrathin nanomesh-reinforced hydrogel.
- Figure S5**.** Optical cross-sections of cold-laminated nanomesh-reinforced hydrogel composites with varying PET thicknesses.
- Figure S6**.** Stress–strain curves of ultrathin nanomesh-reinforced hydrogels with different scaffold densities.
- Figure S7**.**  Stress–strain curves of ultrathin nanomesh-reinforced hydrogels with different PET film thicknesses.
- **Figure S8.** Comparison of Young’s modulus and thickness between this work and the literature.
- **Figure S9.** Digital image of a pig heart with attaching ultrathin nanomesh-reinforced hydrogel based bioelectrode.
- **Figure S10.** Photographs of the ultrathin nanomesh-reinforced hydrogel adhering to a human finger.
- Figure S11**.** Comparison of stress–strain curves for TPU nanomesh, pristine hydrogel, and nanomesh-reinforced hydrogel.
- Figure S12**.** Loading and unloading curves of ultrathin nanomesh-reinforced hydrogel at different strain levels.
- Figure S13**.** Strain-rate-dependent loading–unloading behavior of nanomesh-reinforced hydrogel.
- Figure S14**.** Mechanical hysteresis of nanomesh-reinforced hydrogel under cyclic loading.
- **Figure S15.** SEM images of the ultrathin nanomesh–reinforced hydrogels after 1000 bending cycles.
- Figure S16**.** Comparison of mechanical property of the ultrathin nanomesh-reinforced hydrogel with literature.
- Figure S17**.** Mechanical curves of the pure TPTNa hydrogel with a notch.
- **Figure S18.** Photographs of a notched ultrathin nanomesh-reinforced hydrogel bearing a load and resisting puncture.
- Figure S19**.** Cold-laminated ultrathin alginate hydrogels.
- Figure S20. Digital image of skin covered by ultrathin nanomesh-reinforced hydrogel and PET adhesive tape.
- Figure S21**.** High skin conformability of the ultrathin nanomesh-reinforced hydrogel.
- Figure S22**.** Illustration of the squeezing-induced wrinkle mechanism on skin with different surface coverings.
- **Figure S23.** Comparison of bending circle diameters among different materials.
- **Figure S24.** Digital photographs showing the conformal contact of ultrathin nanomesh-reinforced hydrogel and PET adhesive tape on various objects with different surface textures.
- **Figure S25.** Photographs of the hydrogel showing its appearance at 35 °C, 10 °C, and after reheating to 35 °C.
- **Figure S26.** SEM of hydrogel under high and low temperatures.
- **Figure S27.** Biocompatibility evaluation of ultrathin nanomesh–reinforced hydrogels.
- **Supplementary Note S1.** Mechanism of thermo-responsive adhesion.

**Supplementary Methods**

**Materials.** All chemical reagents used in this research were commercially available and employed directly without any further purification. Acrylic acid (99.0%), N,N’-methylenebisacrylamide (MBA, 99.0%), and sodium sulfate (99%) were purchased from Aladdin Ltd. (Shanghai, China). Tannic acid (98.0%), 2-hydroxy-2-methylpropiophenone (97%), tetrahydrofuran (99.0%), and N,N-dimethylformamide (99.5%) were obtained from Macklin Ltd. (Shanghai, China). N-acryloylglycinamide (NAGA, >98.0%) was sourced from Zhengzhou Alpha Chemical Co., Ltd. PEDOT:PSS (Clevios PH1000) aqueous solution was acquired from Heraeus Co., with a concentration of 1.0–1.3 wt%. Thermoplastic polyurethane (TPU, Elastollan 1185A) was supplied by BASF Co., Ltd. Deionized (DI) water was used for the preparation of all aqueous solutions.

**Preparation of electrospun TPU nanomesh.** The precursor solution was prepared by dissolving 20 wt% TPU pellets in a DMF/THF (1:1) mixture. Electrospinning was carried out using a roller collector connected to the negative pole, rotating at 100 rpm. The applied voltages were set to +10 kV at the positive pole of the high-voltage power supply, with a distance of 15 cm maintained between the two electrodes. The electrospinning process was performed at room temperature under 20%–40% relative humidity for 10, 20, 30, or 35 minutes, with a flow rate of 1 mL/h.

**Preparation of the hydrogel precursor.** The hydrogel was prepared as follows. First, a solution was prepared by mixing 10% (w/w) NAGA, 15% (w/w) AA, and PEDOT at a concentration of 3.75% (v/v) relative to the PEDOT/H₂O solution. Additionally, 0.04% (w/w) N,N′-methylenebisacrylamide (Bis), relative to H₂O, was added. All components were dissolved in 10 mL of double-distilled water to obtain a homogeneous solution. Subsequently, Na₂SO₄ (0.2 M relative to the Na₂SO₄/H₂O solution), TA (1.5% w/w relative to the TA/H₂O solution), and the photoinitiator I1173 (12 μL) were introduced to form the hydrogel precursor. After stirring for 5 minutes, the precursor solution was subjected to UV irradiation (365 nm) for 2 minutes to increase its viscosity and generate the hydrogel precursor.

**Preparation of ultrathin composite hydrogels.** Building upon the electrospun TPU nanofibrous membrane, ultrathin composite hydrogels were fabricated through a controlled coating process. A precise volume of hydrogel precursor solution was aspirated using a micropipette and evenly spread onto the surface of silicone-coated paper bearing the electrospun membrane. Subsequently, a PET film was carefully placed over the precursor solution to facilitate uniform film formation. To ensure precise control of the hydrogel thickness, the roller gap of a laminator was adjusted to a predetermined distance, allowing excess precursor solution to be extruded by the rotating rollers and distributed uniformly across the nanofibrous membrane. The final thickness of the resulting composite film was regulated by the thickness of the PET film. Following film formation, the composite membrane was exposed to UV light (365 nm) for 1 minute to induce photo-crosslinking and achieve solidification. After crosslinking, the PET support layer was promptly removed, yielding a freestanding ultrathin nanofibrous composite hydrogel. To further optimize the hydrogel composition and film-forming performance, control samples with varying electrospinning durations and PET film thicknesses were prepared using the same protocol. Detailed experimental parameters and characterization results are summarized in **Table S1**.

**General characterization.** The morphology of hydrogels was observed using a cryo-scanning electron microscope (Cryo-SEM, FEI Quanta 450). Chemical composition of hydrogels were investigated using Raman spectroscopy (WITec alpha300 R), and Fourier transform infrared spectrometer (FTIR, Nicolet iS10).

**Mechanical property assessment.** Mechanical tests of the hydrogel samples were conducted using a universal testing machine (ZQ-990LB, 500 N, China) at a constant stretching rate of 100 mm/min. Standard rectangular specimens measuring 30 mm in length and 10 mm in width were prepared for the tensile tests, with a clamping distance set to 8 mm.

**Adhesion separation test.** An L-shaped acrylic holder (2.5 cm × 4 cm) was used to secure the samples, with an initial contact area of 1 cm × 1 cm between the nanonet-supported hydrogel and the test substrates. The holder was lifted vertically at a constant rate of 60 mm min⁻¹ until the hydrogel was completely separated from the substrate. During this process, the adhesion force and displacement were recorded using a universal testing machine (ZQ-990LB, 500 N, China). The adhesion energy was calculated by dividing the peak adhesion force by the contact area.

**Conductive property measurement.** The conductivity of the hydrogels was determined using a four-electrode AC impedance method on an electrochemical workstation (CHI660E, China). During the measurements, an AC voltage of 0.1 V was applied, with frequencies ranging from 0.1 Hz to 10^5^ Hz.

**Cytocompatibility test.** L929 fibroblasts (L929) were employed to assess the cytotoxicity of the hydrogels. For live/dead staining, first, L929 cells in the logarithmic growth phase were digested with trypsin to prepare a cell suspension. The cell suspension concentration was adjusted, and cells were seeded into six-well plates at a density of 4×10^5 cells/well and cultured for 24 hours to allow for cell attachment. Next, according to the experimental groups, 0% and 10% hydrogel extract were added to the respective groups of cells for 24 hours and 48 hours. Finally, following the reagent kit instructions, a 1× working solution (Calcein-AM/PI, C2015S, Biyuntian) was prepared using the HBSS buffer. The culture medium was removed from the six-well plates, washed three times with HBSS, and the prepared Calcein-AM/PI working solution was added to the cells. The cells were incubated at 37°C for 30 minutes, and after 30 minutes, photographs were taken using a fluorescence microscope (MF53, Guangzhou Mingmei Optoelectronics Technology Co., Ltd., China).

**Water vapor transmission rate (WVTR) measurement.** The water vapor transmission rate (WVTR) was measured using a gravimetric method. Three identical containers were filled with the same initial mass of water. Prior to the experiment, the masses of the empty container, the hydrogel membrane, and the TPU film were individually recorded. During the test, membranes with identical dimensions—ultrathin hydrogel membrane, TPU film, or no covering (blank control)—were placed over the container opening. The total mass of the system, including the container, covering material, and remaining water, was recorded daily. To independently monitor the hydration stability of the hydrogel, an additional hydrogel sample with the same dimensions was prepared and weighed daily in parallel. These measurements were used solely to assess hydrogel mass stability and were not included in the WVTR calculation. At each time point, the mass of water remaining in the container was determined by subtracting the pre-recorded masses of the container and the corresponding covering material from the total measured mass. The decrease in water mass over time was attributed to water vapor transmission through the membrane. The WVTR was calculated according to the following equation

$$\mathrm{WVTR}= \frac{\Delta m}{A \times\Delta t}$$

where $\Delta m$ is the mass loss of water from the container over the time interval $\Delta t$, and $A$ is the effective exposed area of the covering membrane.

**Applications in epidermal electrodes.** Electrocardiogram (ECG) signals were acquired using both the hydrogel electrodes and commercial Ag/AgCl electrodes, connected to a biosignal acquisition system (ADS1292, Wuxi Xinweilai Technology Co., Ltd.). To record ECG signals, two circular hydrogel electrodes with a diameter of 1 cm were symmetrically attached to the inner wrists of the volunteer’s right and left arms. An additional hydrogel electrode was affixed to the inner right ankle to serve as the ground electrode. The signal-to-noise ratio (SNR) was calculated using the equation:

SNR=20lg (Amp/Std)

where Amp represents the amplitude of the ECG signal, and Std is the standard deviation of the baseline signal measured between 0.2 s and 0.3 s prior to the peak ECG amplitude.

**Signal preprocessing and time–frequency analysis.** The raw signals were first segmented using a sliding-window approach with a window length of 1000 samples and a step size of 100 samples, generating partially overlapping segments to capture local temporal characteristics. Each segment was subsequently transformed into the time–frequency domain using the short-time Fourier transform (STFT). The STFT was performed using a Hamming window of 16 samples with a 50% overlap (8 samples) and 1000 FFT points at a sampling frequency of 120 Hz. The magnitude of the STFT coefficients was converted to a logarithmic scale (20·log₁₀|S|) to generate spectrogram representations. All spectrograms were visualized using a consistent colormap and resized to 64 × 64 pixels to ensure uniform input dimensions for subsequent analysis.

**In vivo arrhythmia induction experiment.** In vivo arrhythmia induction was performed by tail-vein injection of aconitine. Briefly, the distal one-third of the rat tail was wiped with 75% ethanol to facilitate visualization of the lateral tail vein. A 1 mL syringe was used to draw a 0.002% aconitine solution, which was administered at a dosage of 1.25 mL kg⁻¹ body weight. Either the left or right lateral tail vein was selected for injection. The needle was inserted into the vein at an angle of approximately 10°–15°, and after skin penetration, the needle tip was advanced parallel to the vein. Successful venous access was confirmed by blood backflow, after which the aconitine solution was injected steadily and completed within 5 s. Electrocardiogram (ECG) recording was initiated immediately after aconitine administration to capture the onset and progression of arrhythmia.


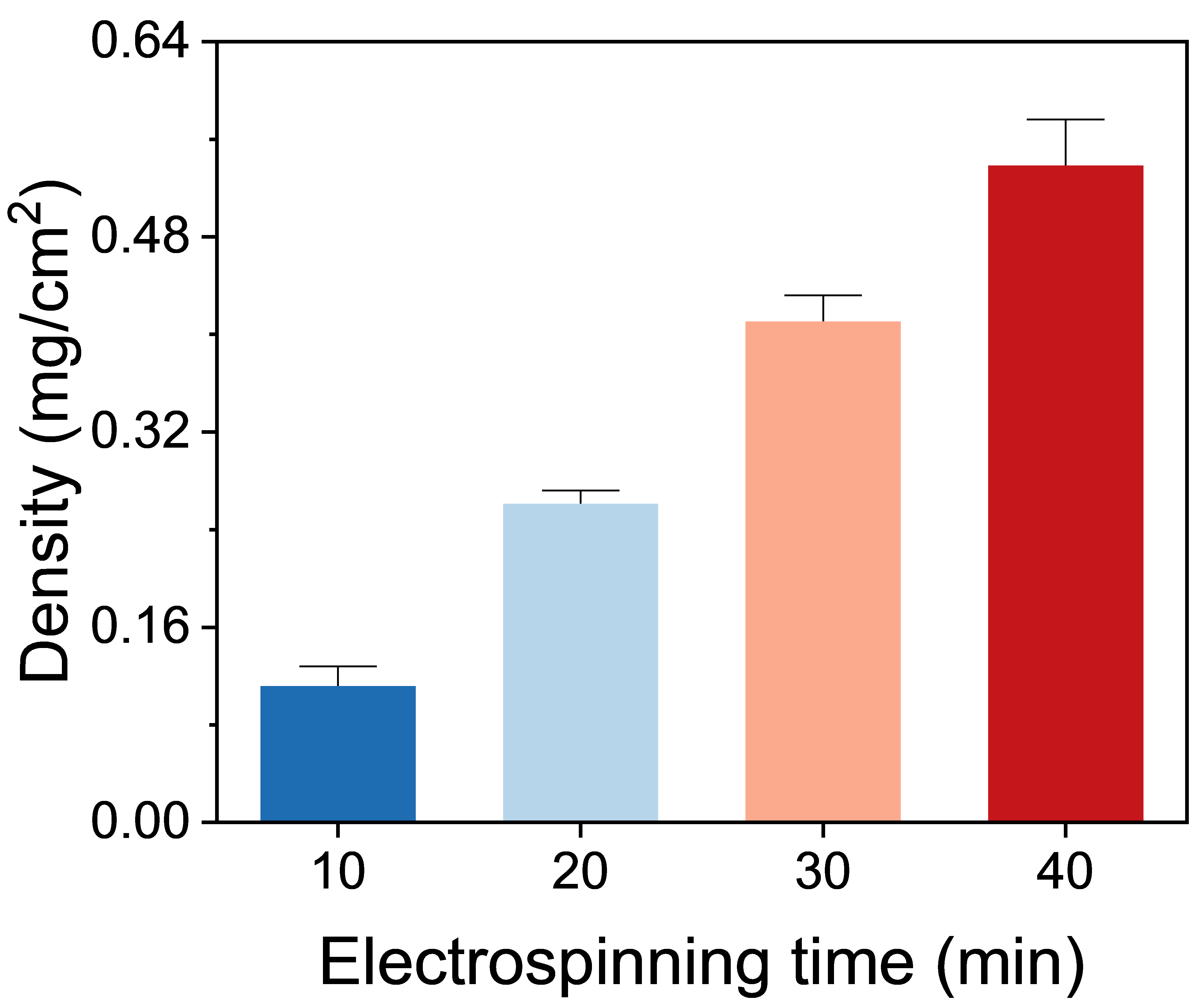


Figure S1**.** Effect of electrospinning duration on nanofiber mat density.


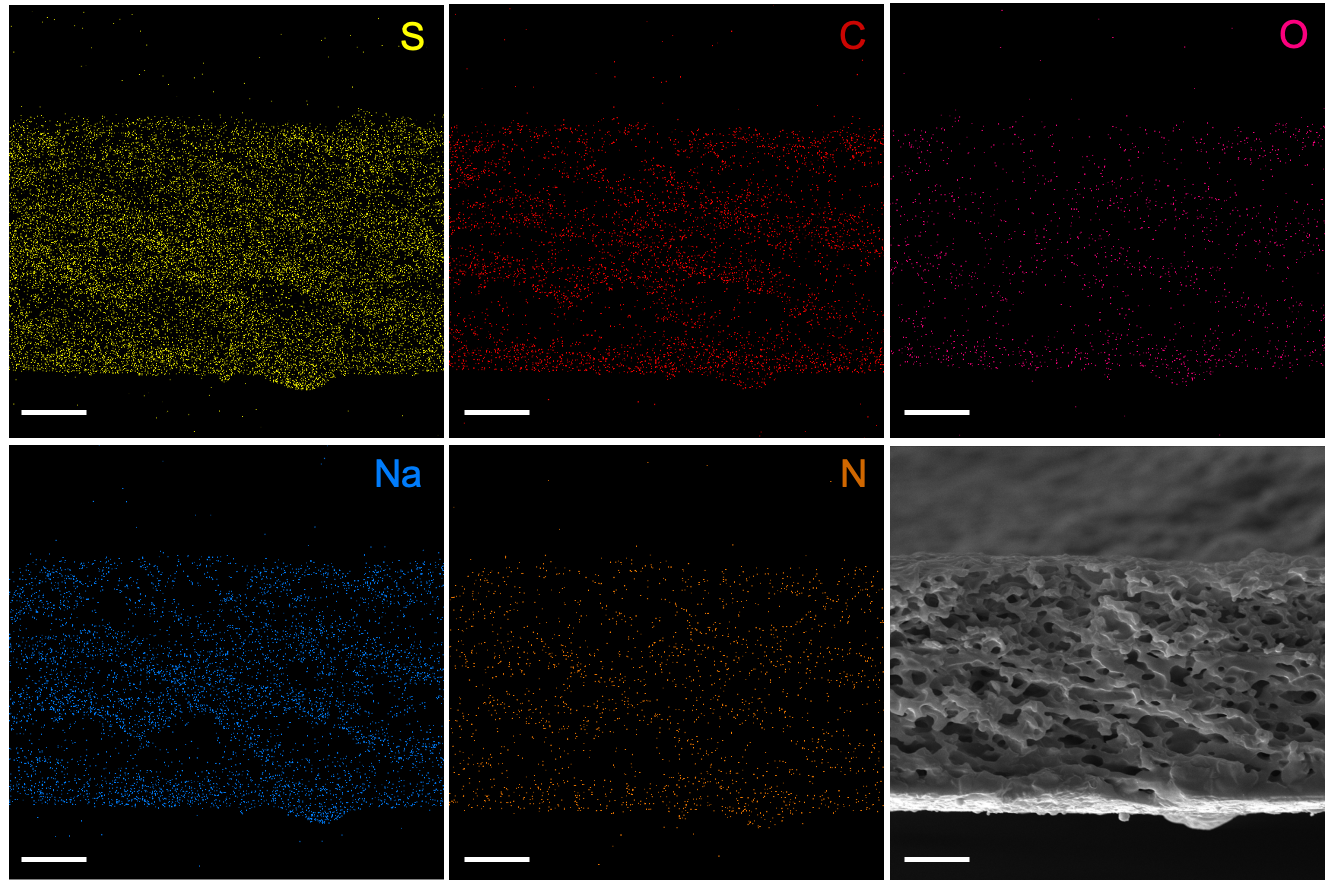


Figure S2**.** Cross-sectional mapping of the ultrathin nanomesh-reinforced hydrogel (scale bar = 20 µm).


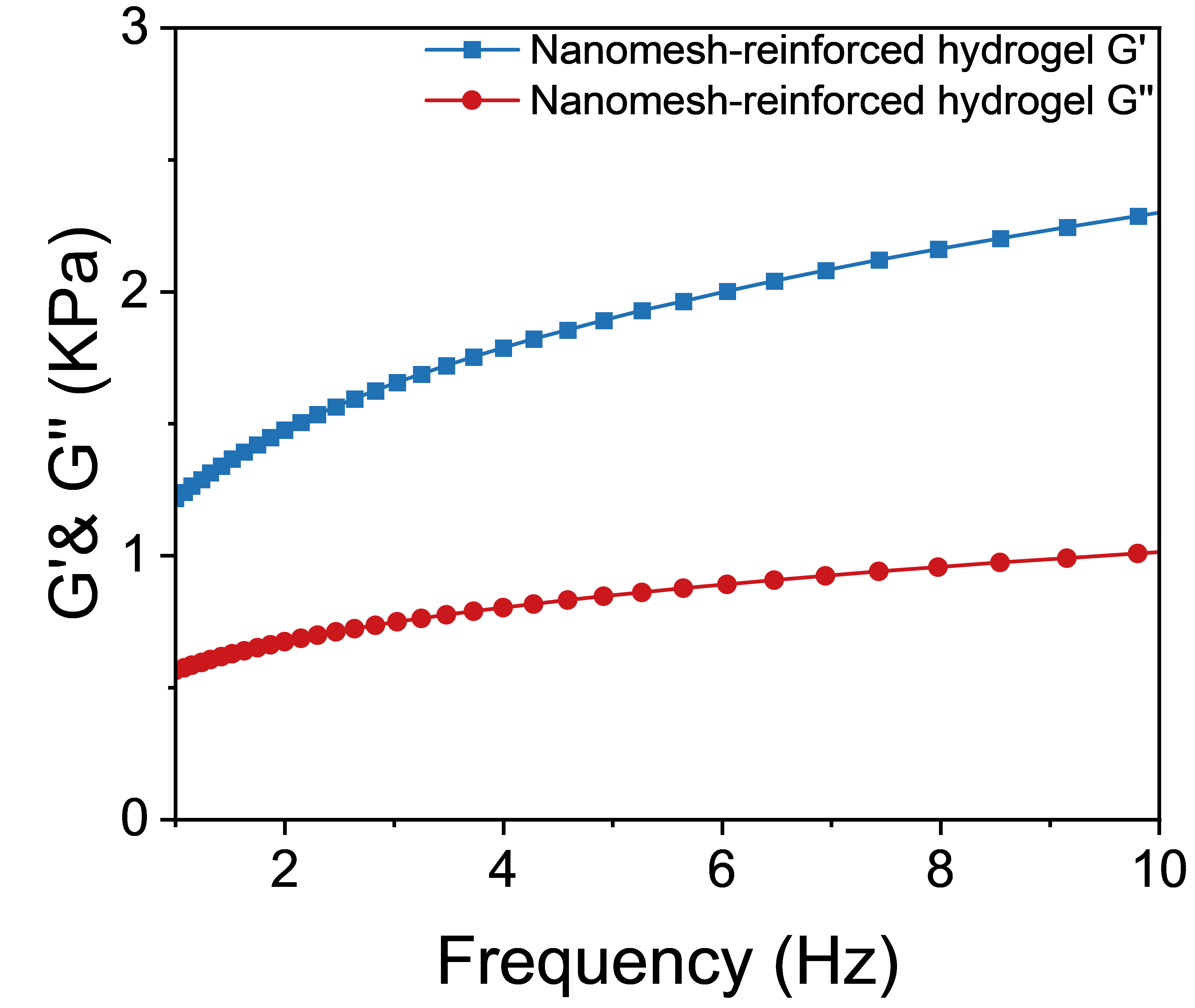


Figure S3**.** Rheological properties of the ultrathin nanomesh-reinforced hydrogel.


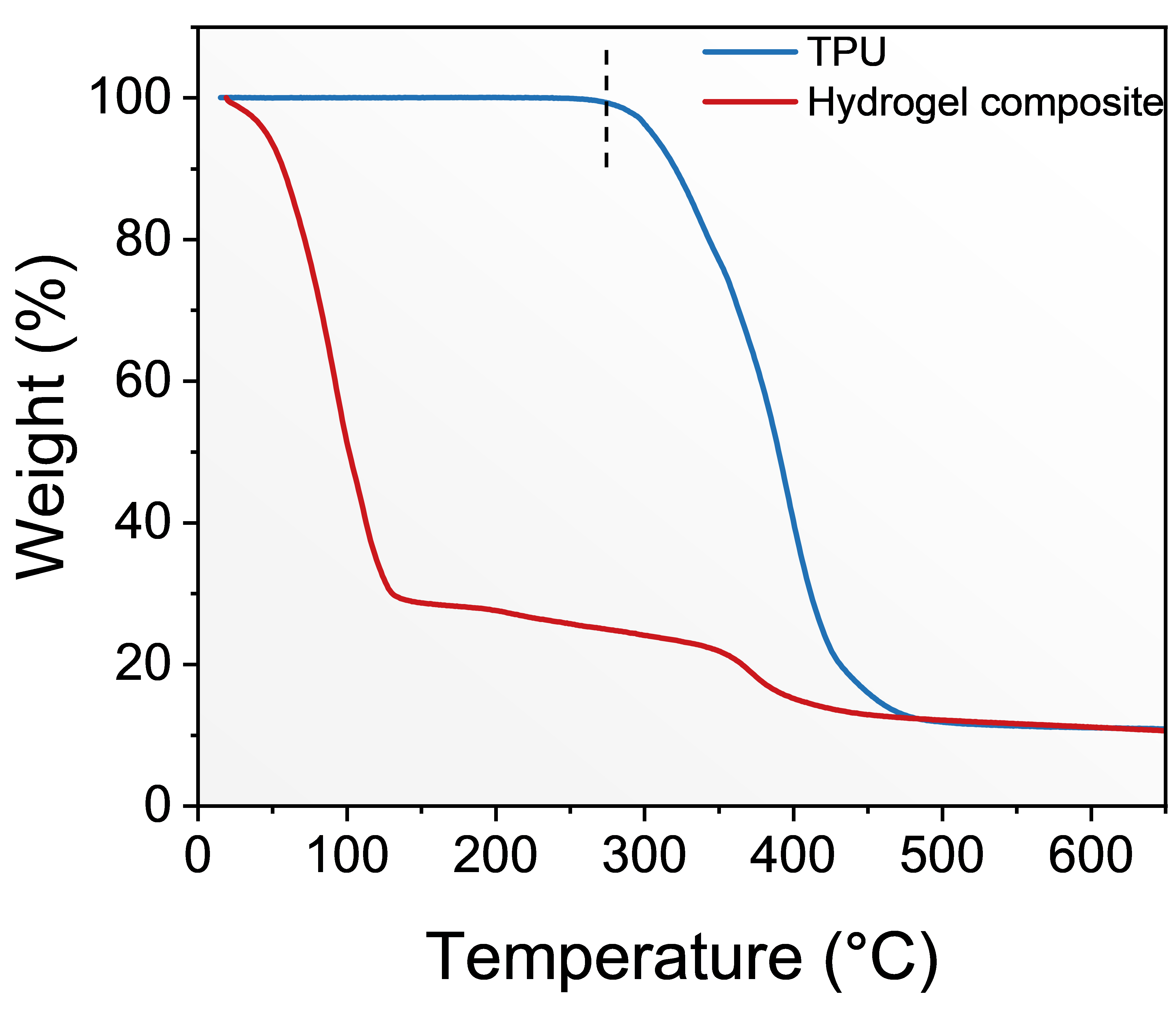


Figure S4. TGA curves of TPU nanomesh scaffold and ultrathin nanomesh-reinforced hydrogel.

**Table S1.** Composition of the nanomesh-reinforced hydrogel.

| **Sample code** | **Electrospinning time (min)** | **PET Thickness (μm)** |
| --- | --- | --- |
| 1 | 10 | 75 |
| 2 | 20 | 75 |
| 3 | 30 | 75 |
| 4 | 40 | 75 |
| 5 | 20 | 25 |
| 6 | 20 | 50 |
| 7 | 20 | 75 |
| 8 | 20 | 100 |


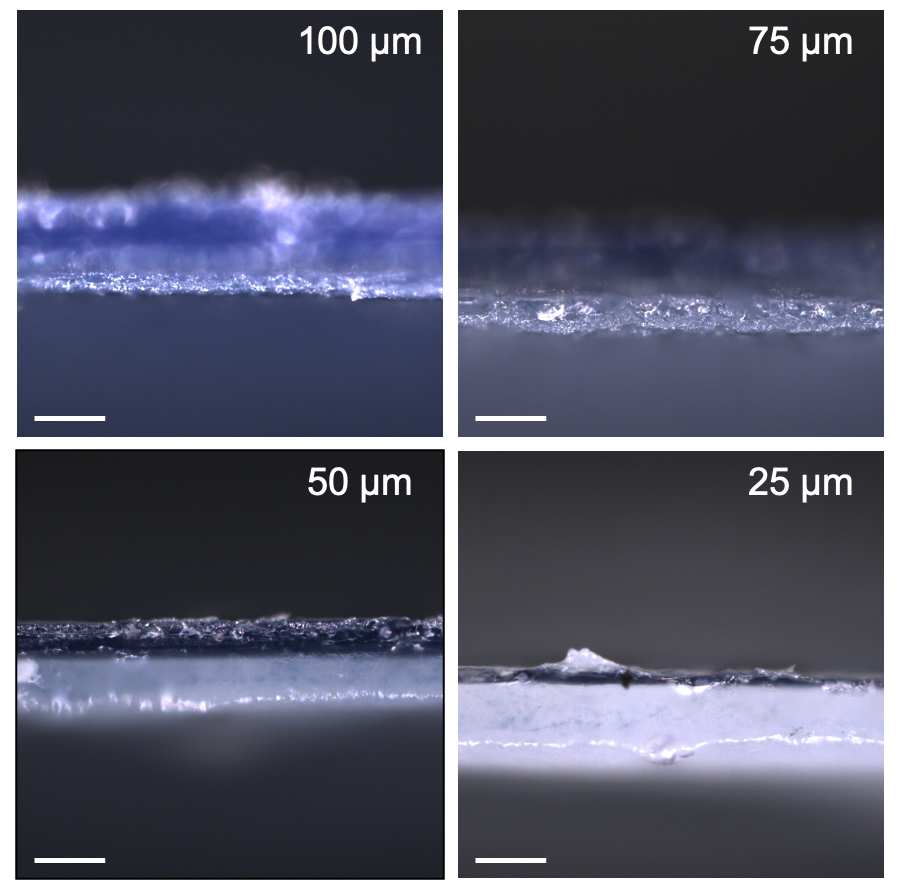


Figure S5**.** Optical cross-sections of cold-laminated nanomesh-reinforced hydrogel composites with varying PET thicknesses (scale bar = 100 µm)


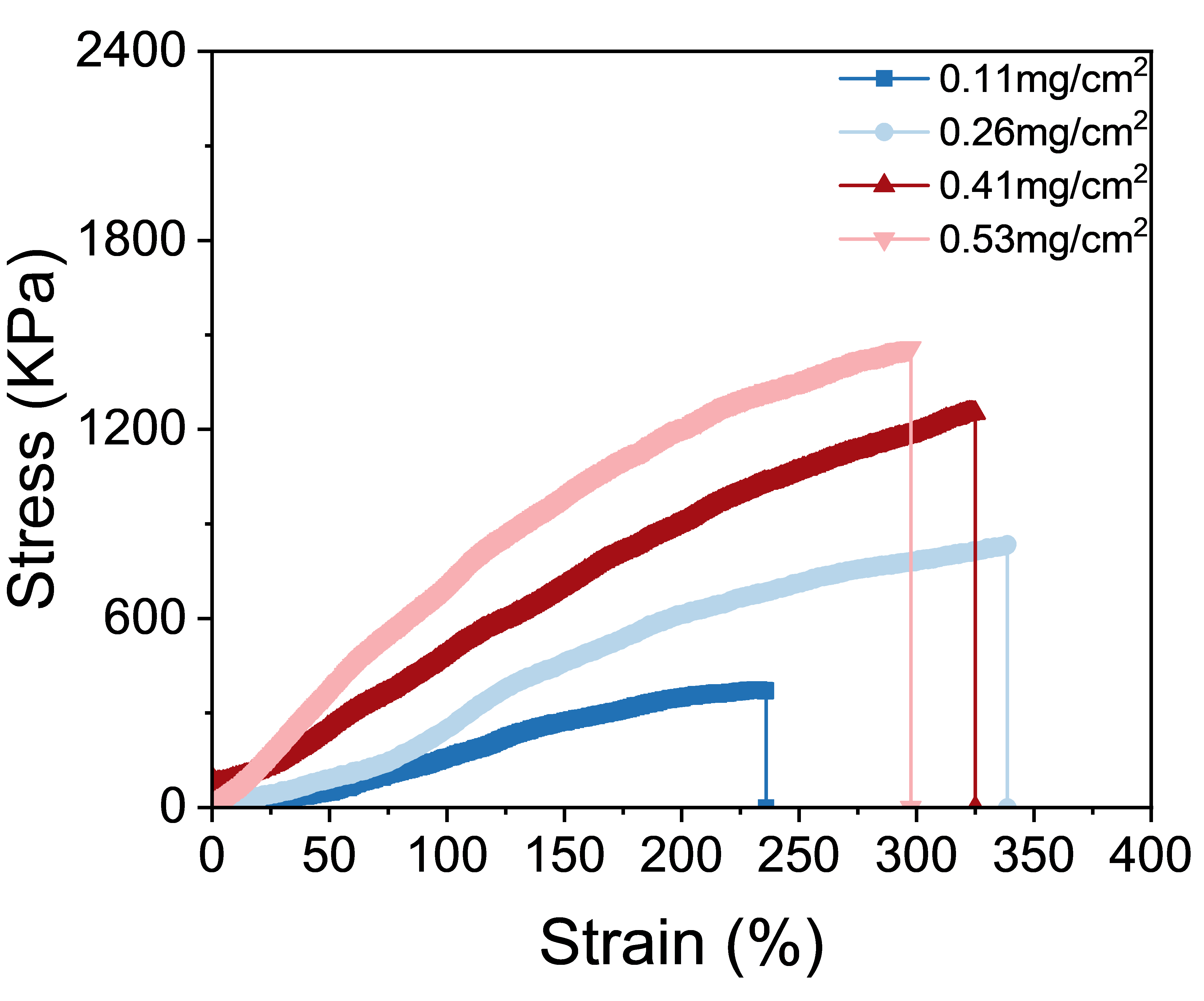


Figure S6**.** Stress–strain curves of ultrathin nanomesh-reinforced hydrogels with different scaffold densities


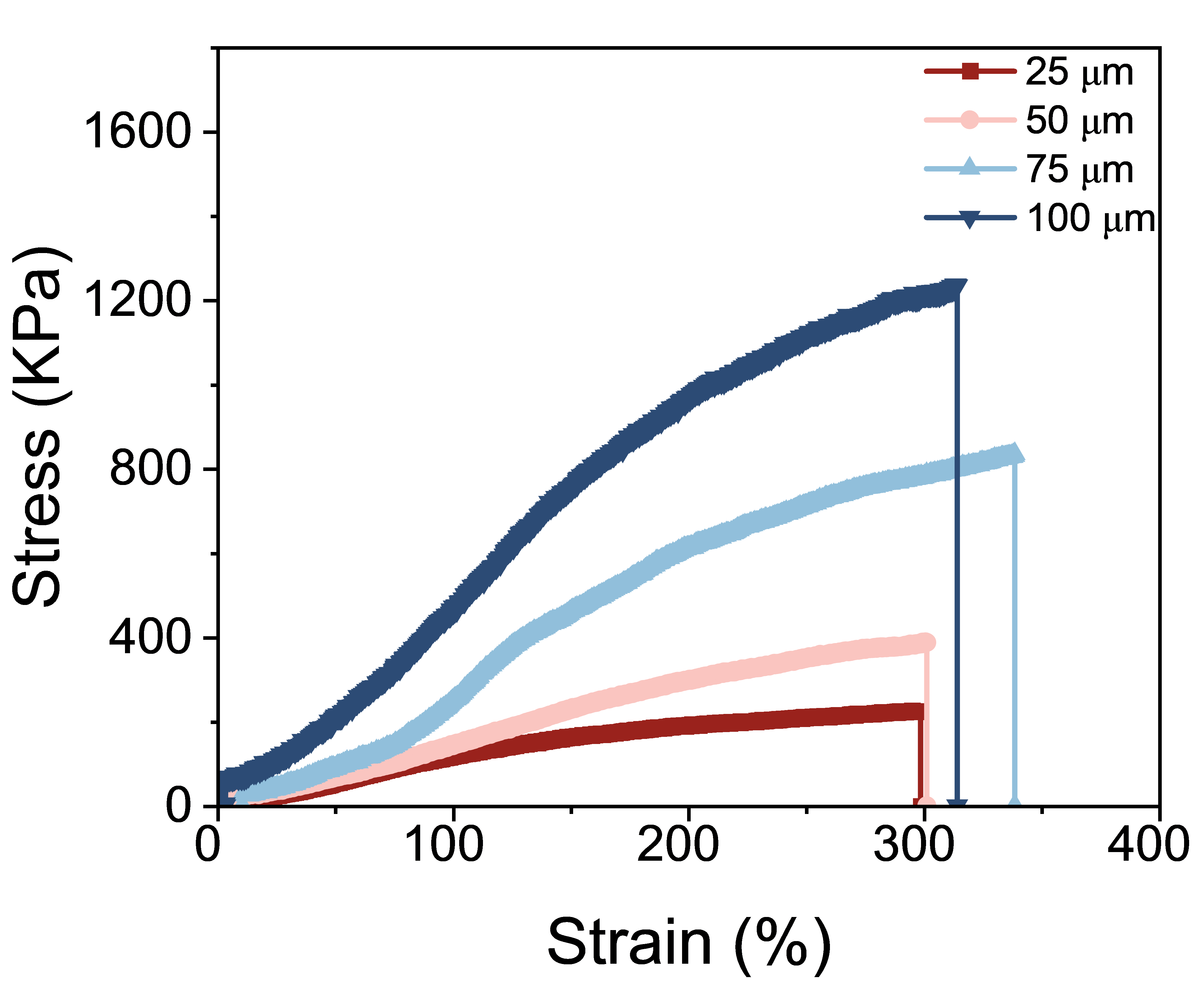


Figure S7**.**  Stress–strain curves of ultrathin nanomesh-reinforced hydrogels with different PET film thicknesses.

**Table S2.** Young’s moduli and tensile strain of ultrathin nanomesh-reinforced hydrogel with different thicknesses.

| **Thickness**  **(μm)** | **Electrospinning time (min)** | **Young’s modulus**  **(kPa)** | **Elongation at break (%)** | **Toughness**  **(KJ/m^3^)** |
| --- | --- | --- | --- | --- |
| 17 | 20 | 324.7± 4.3 | 314.1 | 1765.62 |
| 40 | 10 | 35.3 ± 2.1 | 234.4 | 461.71 |
| 40 | 20 | 173.4 ± 2.3 | 338.7 | 1570.27 |
| 40 | 30 | 367.1 ± 3.8 | 325.1 | 2331.45 |
| 40 | 40 | 761.1 ± 3.1 | 297.2 | 2641.72 |
| 62 | 20 | 110.3 ± 1.1 | 300.5 | 551.99 |
| 90 | 20 | 92.3 ± 1.1 | 298.7 | 426.35 |


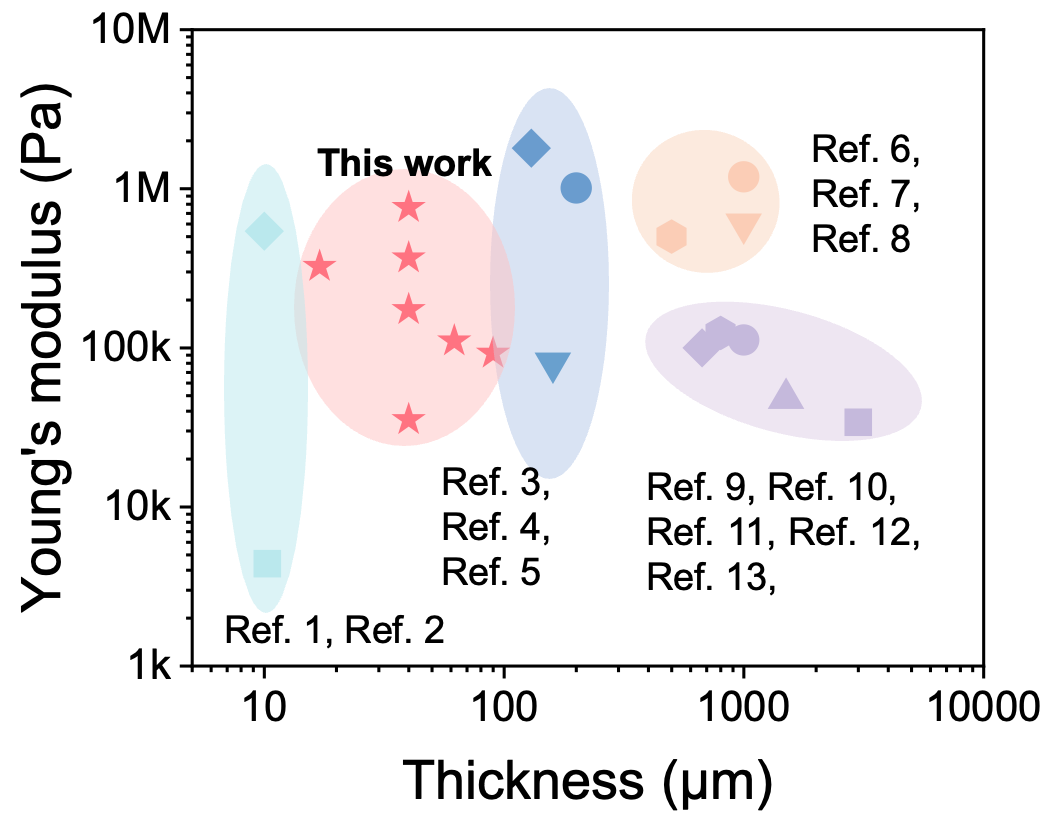


**Figure S8.** Comparison of Young’s modulus and thickness between this work and the literature. [1, 2] [3-5] [6-8] [9-13]


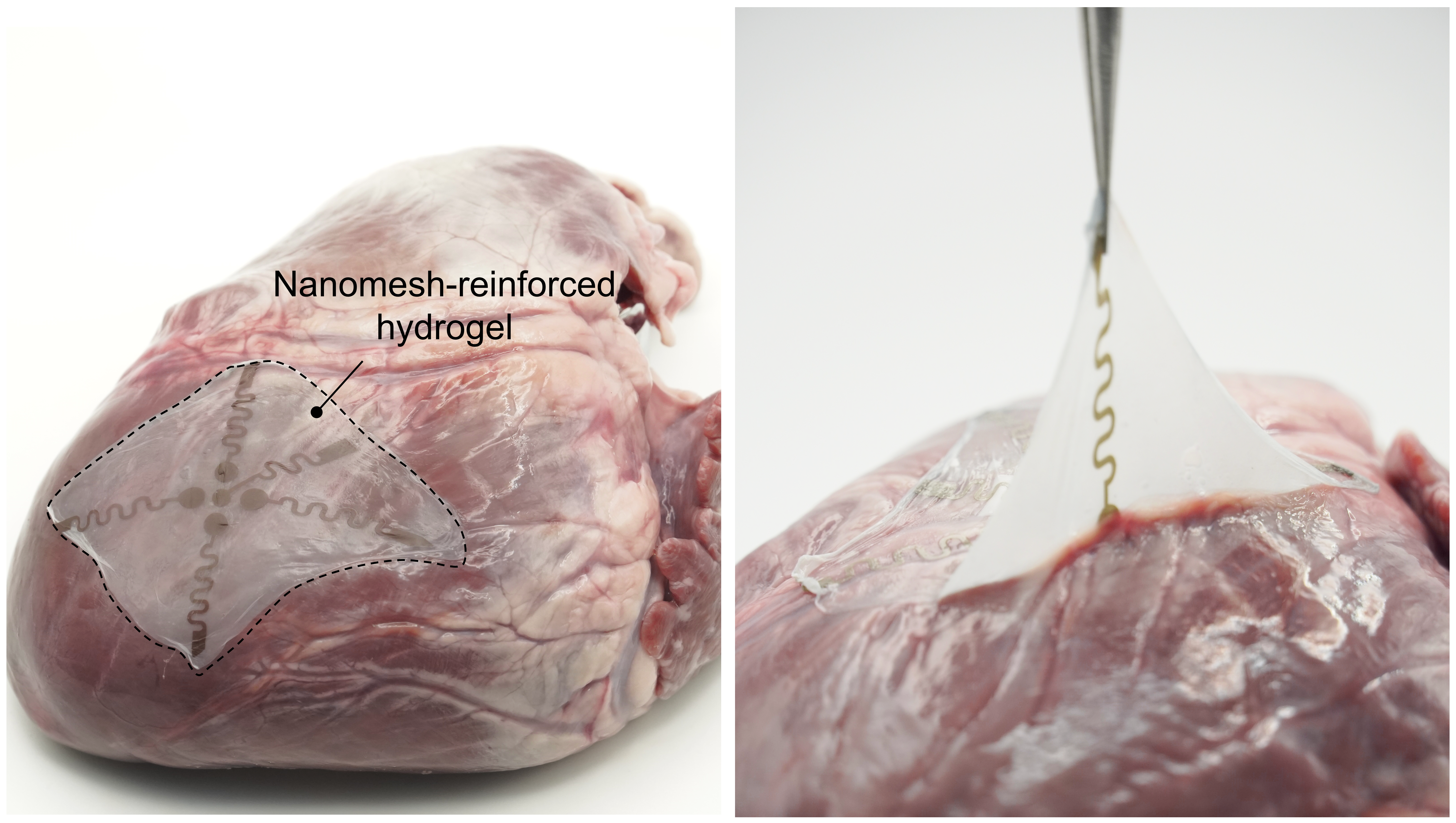


**Figure S9.** Digital image of a pig heart with attaching ultrathin nanomesh-reinforced hydrogel based bioelectrode.


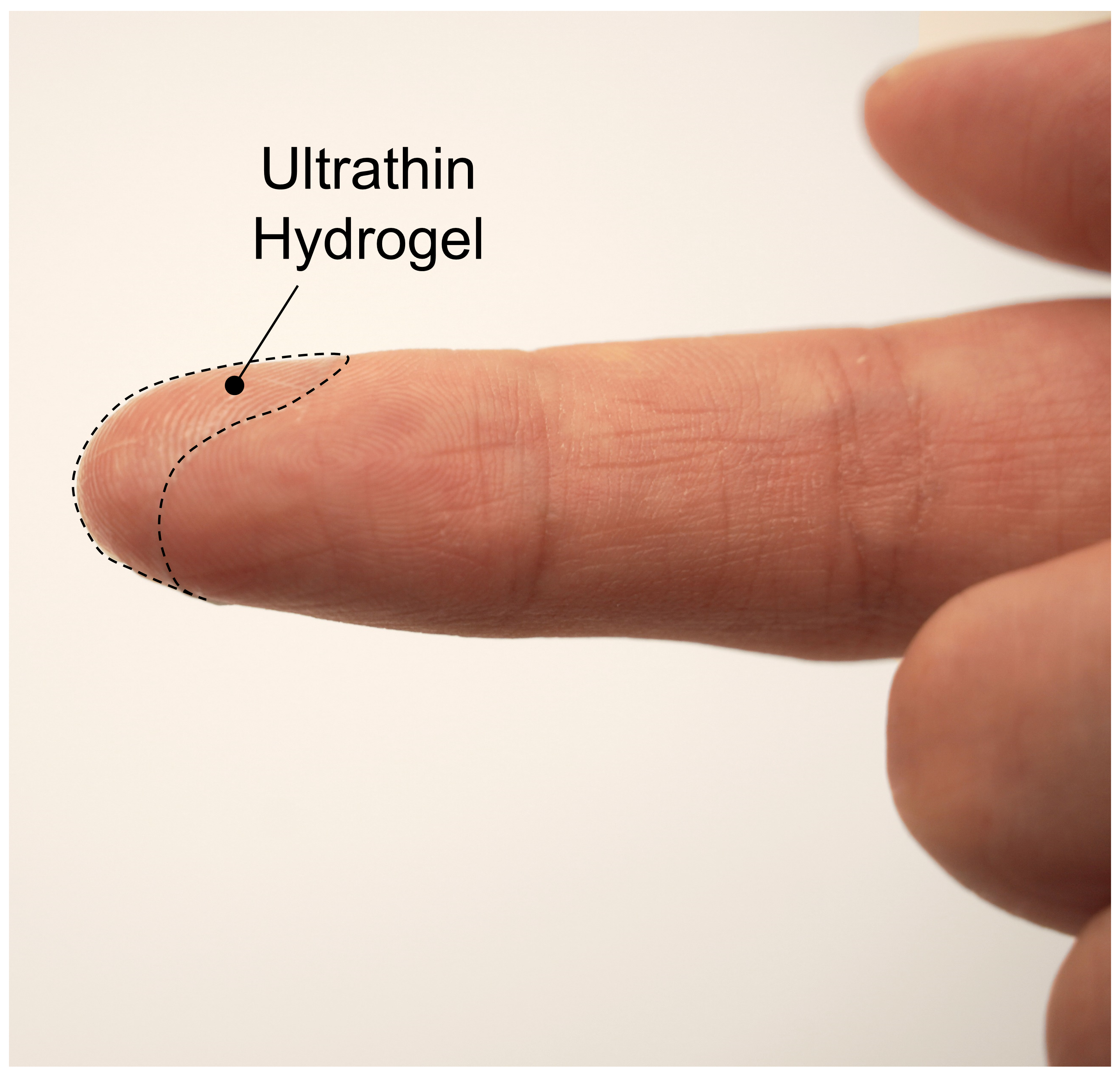


**Figure S10.** Photographs of the ultrathin nanomesh-reinforced hydrogel adhering to a human finger.


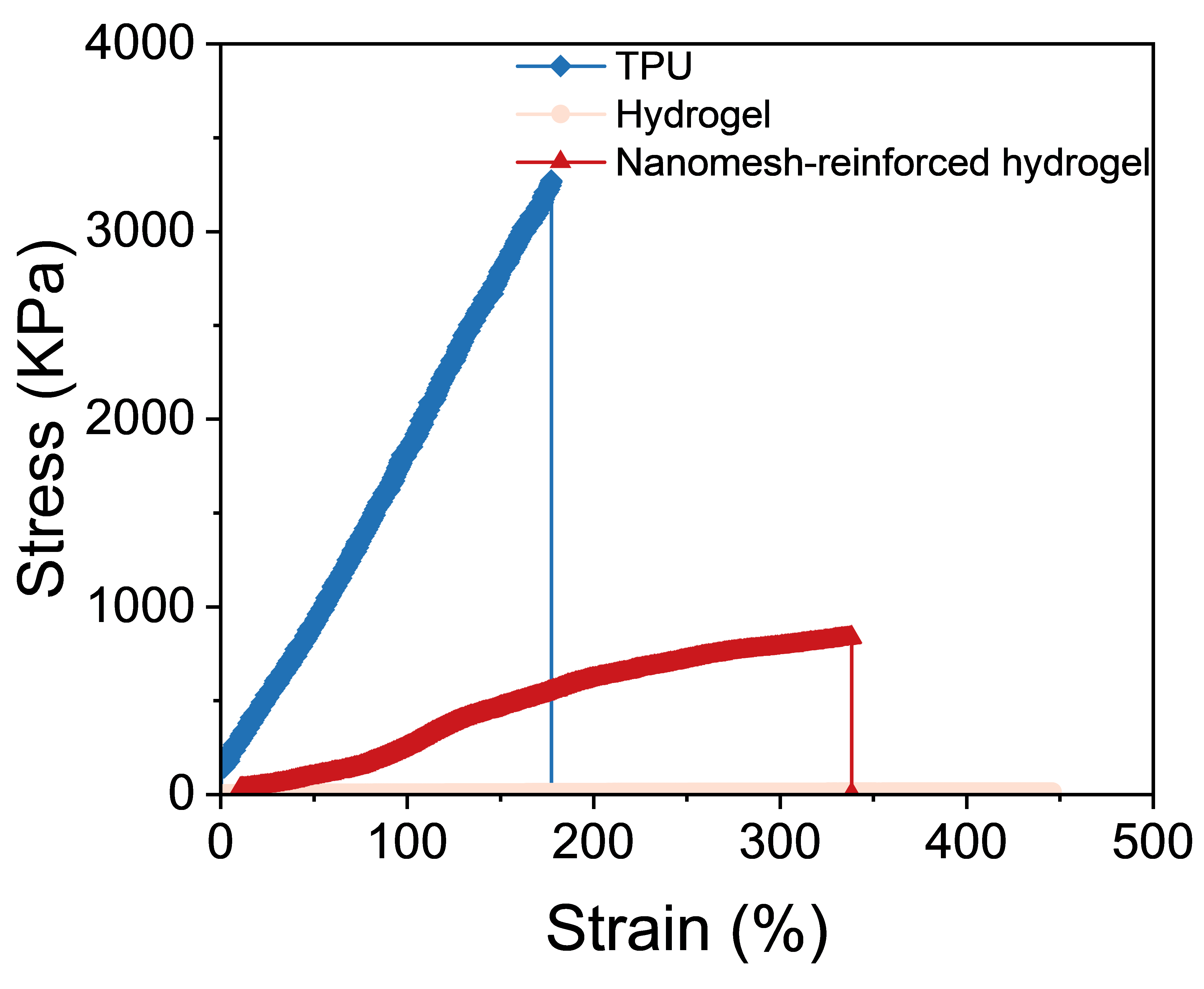


Figure S11**.** Comparison of stress–strain curves for TPU nanomesh, pristine hydrogel, and nanomesh-reinforced hydrogel.


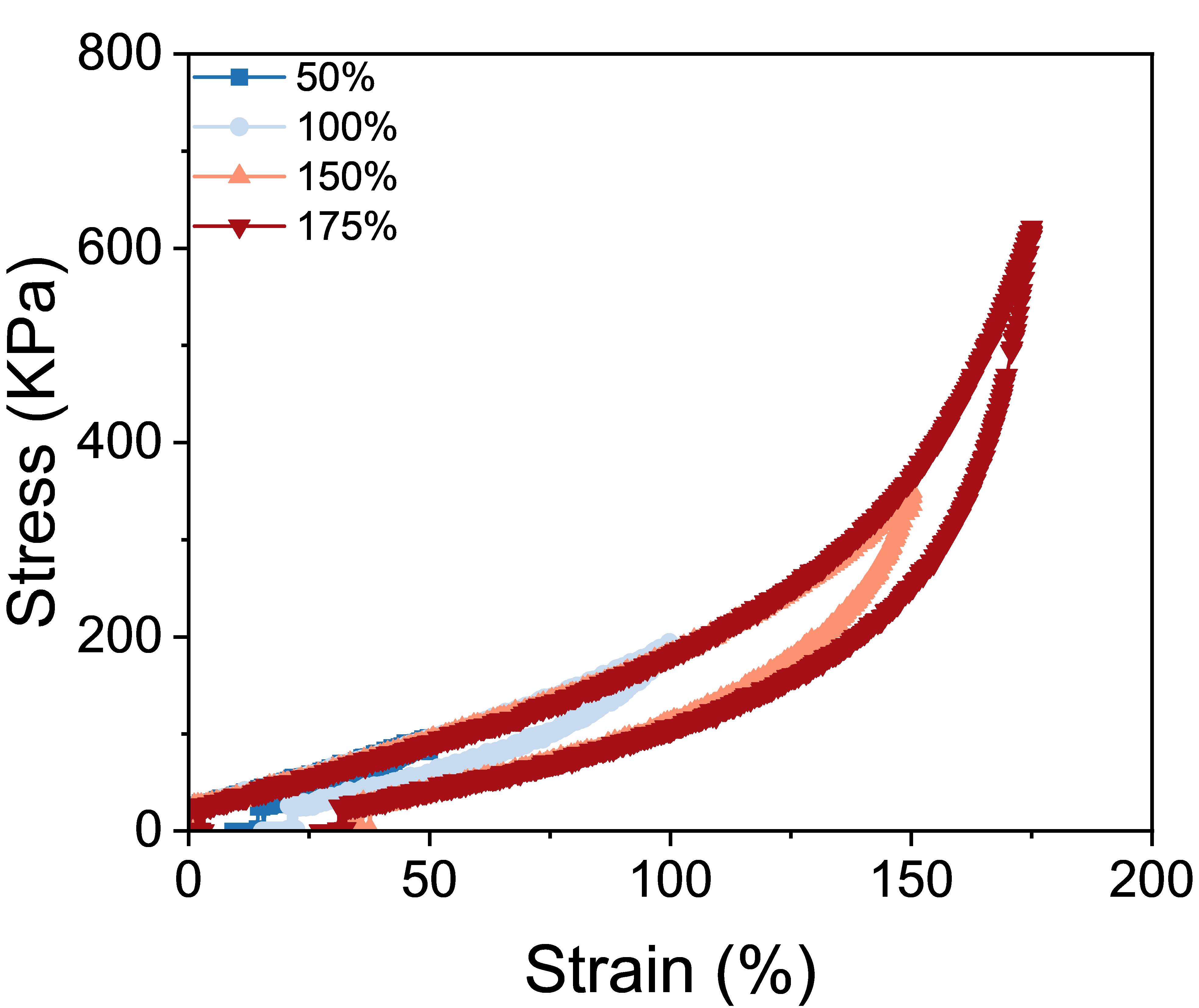


Figure S12**.** Loading and unloading curves of ultrathin nanomesh-reinforced hydrogel at different strain levels


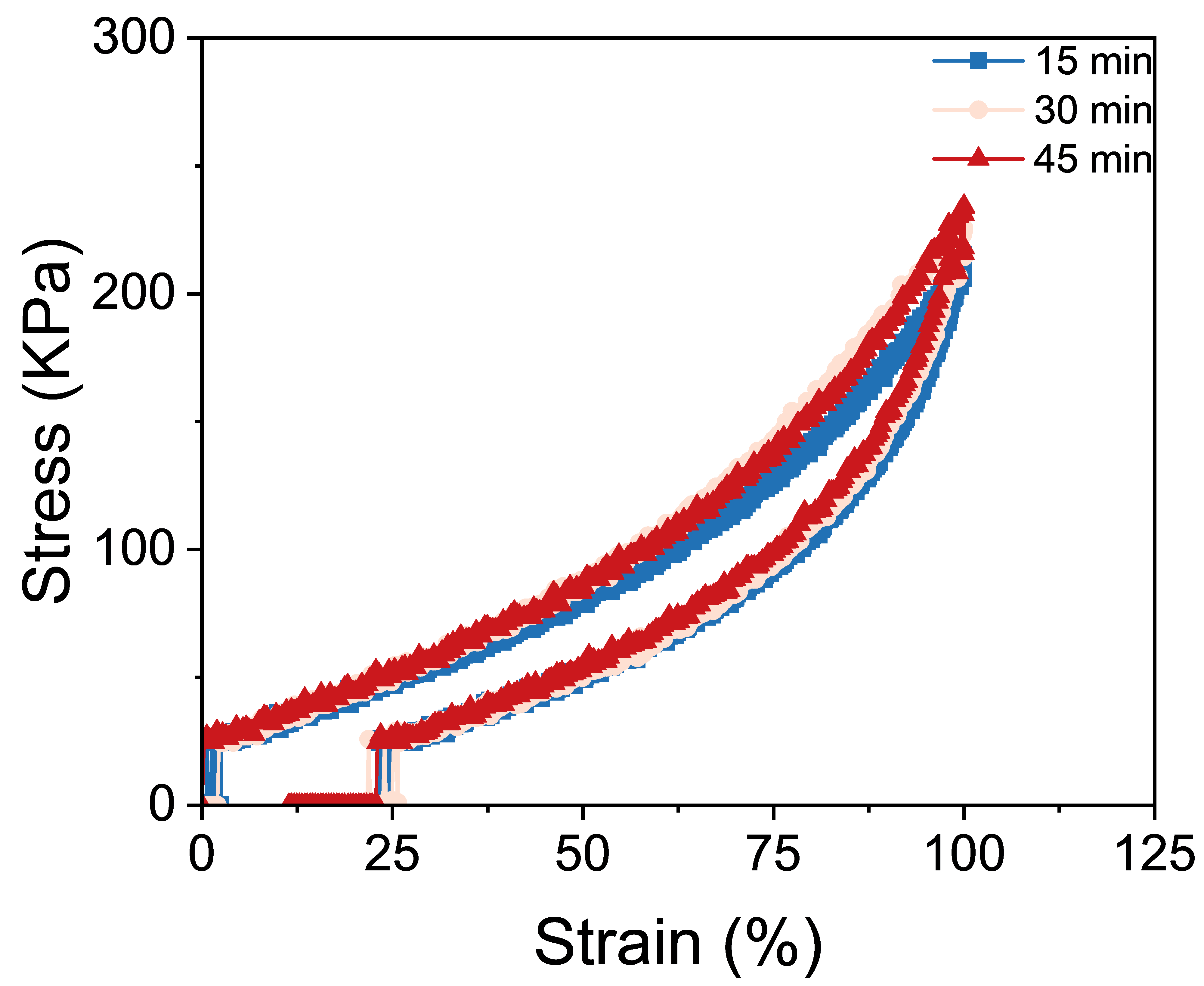


Figure S13**.** Strain-rate-dependent loading–unloading behavior of nanomesh-reinforced hydrogel.


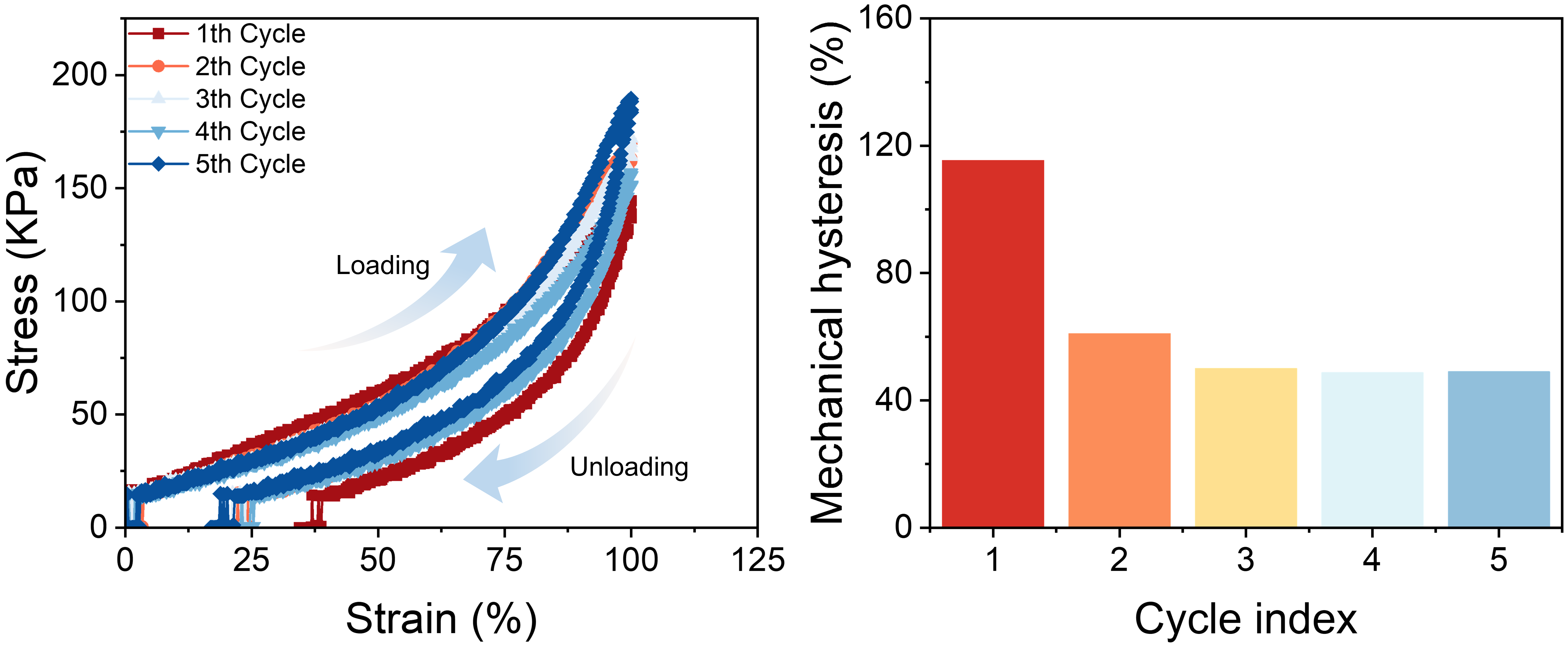


Figure S14**.** Mechanical hysteresis of nanomesh-reinforced hydrogel under cyclic loading.


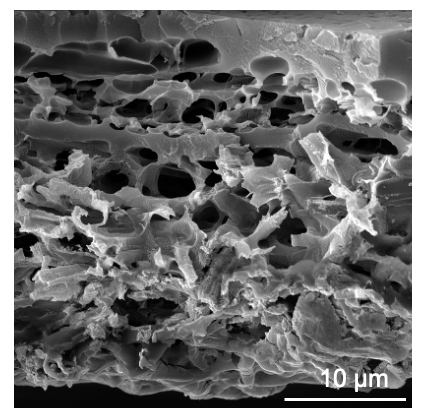


Figure S15**.** SEM images of the ultrathin nanomesh–reinforced hydrogels after 1000 bending cycles.


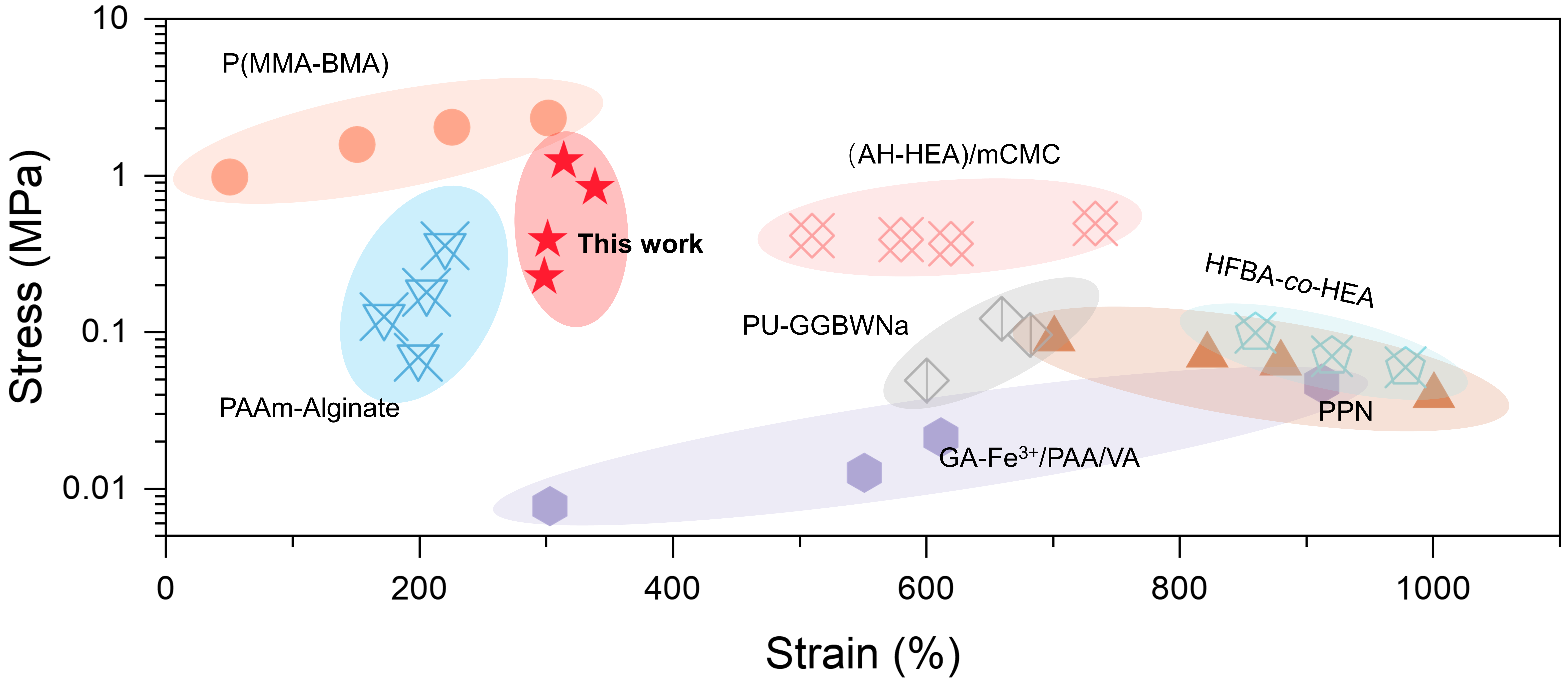


Figure S16**.** Comparison of mechanical property of the ultrathin nanomesh-reinforced hydrogel with literature.

**Table S3.** Performance comparison between ultrathin nanomesh-reinforced hydrogel and reported flexible electronic skin sensors.

| **Material** | **Thickness**  **(**µm**)** | **Toughness** | **Stress** | **Application** | **Ref** |
| --- | --- | --- | --- | --- | --- |
| PU nanomesh/Gelatin/  Glycerol/Borax/Na₂SO₄ | 10.3 | 7.3 ±1 KJ/m^3^ | ~50 KPa | ECG/EMG/  EEG/EOG | [2] |
| PU microfiber/PVA/MF  /Glycerol-CH hydrogel | ~5 | / | ~6 MPa | EMG | [4] |
| P(HFBA-co-HEA)/  [EMIM][TFSI] ionogel | 1600 | / | ～60 KPa | Strain sensor | [14] |
| P(AH-HEA)/mCMC hydrogel | 2000 | 3.96 MJ/m^3^ | 0.58 MPa | Strain sensor | [15] |
| Gallic acid/Fe³⁺-H₂O₂ hydrogel | / | 270 KJ/m^3^ | ～50 KPa | Transdermal drug-delivery motion | [16] |
| MXene-CNTs-TPU film | / | / | ～0.9 KPa | ECG/EMG | [17] |
| MXene-Zr⁴⁺/  gelatin/PAAc hydrogel | 1500 | / | 1.5 MPa | Underwater  sensor | [18] |
| Polypyrrole/PAAm-IL-Li/CsPbBr₃ hydrogel | 1000 | / | ~175 KPa | Motion detection | [19] |
| [VBIm][BF₄]/[C₁₂VIm]  [BF₄]/[BMIm][BF₄] ionogel | 1000 | / | 4.5 MPa | Motion detection | [20] |
| Gelatin/PPy/rGO organohydrogel | 3000 | ~210 KJ/m^3^ | 50 KPa | Motion detection  /ECG/EMG | [13] |
| Sodium alginate/PAM/  PEDOT:PSS hydrogel | 2000 | / | ~10 KPa | EEG | [21] |
| Graphene/PVA/PVP hydrogel | 1000 | / | ~550 KPa | Motion detection | [11] |
| TPU nanonet/  [EMIM][TFSI] ionogel | 16.7 | 5.51 MJ/m^3^ | 2.78 MPa | Motion detection  /ECG/EMG  /EOG/EEG | [22] |
| Cellulose/Zn^2+^/Ca^2+^/ Glycerol | / | 37.5 KJ/m^3^ | ~280 KPa | Anti-freezing | [23] |
| PAAm/PVA/NaCl/  Glycerol | / | ~1410 KJ/m^3^ | 120.7 KPa | Motion detection | [24] |
| LM/GO/PEDOT/  PAM/PDA | 5000 | 903.82 KJ/m^3^ | 56 KPa | Motion detection | [25] |
| **PU nanomesh/NAGA/**  **AA/TA/PEDOT:PSS/**  **Na_2_SO_4_ hydrogel** | **17** | **2641.72**  **KJ/m^3^** | **1459 KPa** | **ECG** | **This**  **work** |


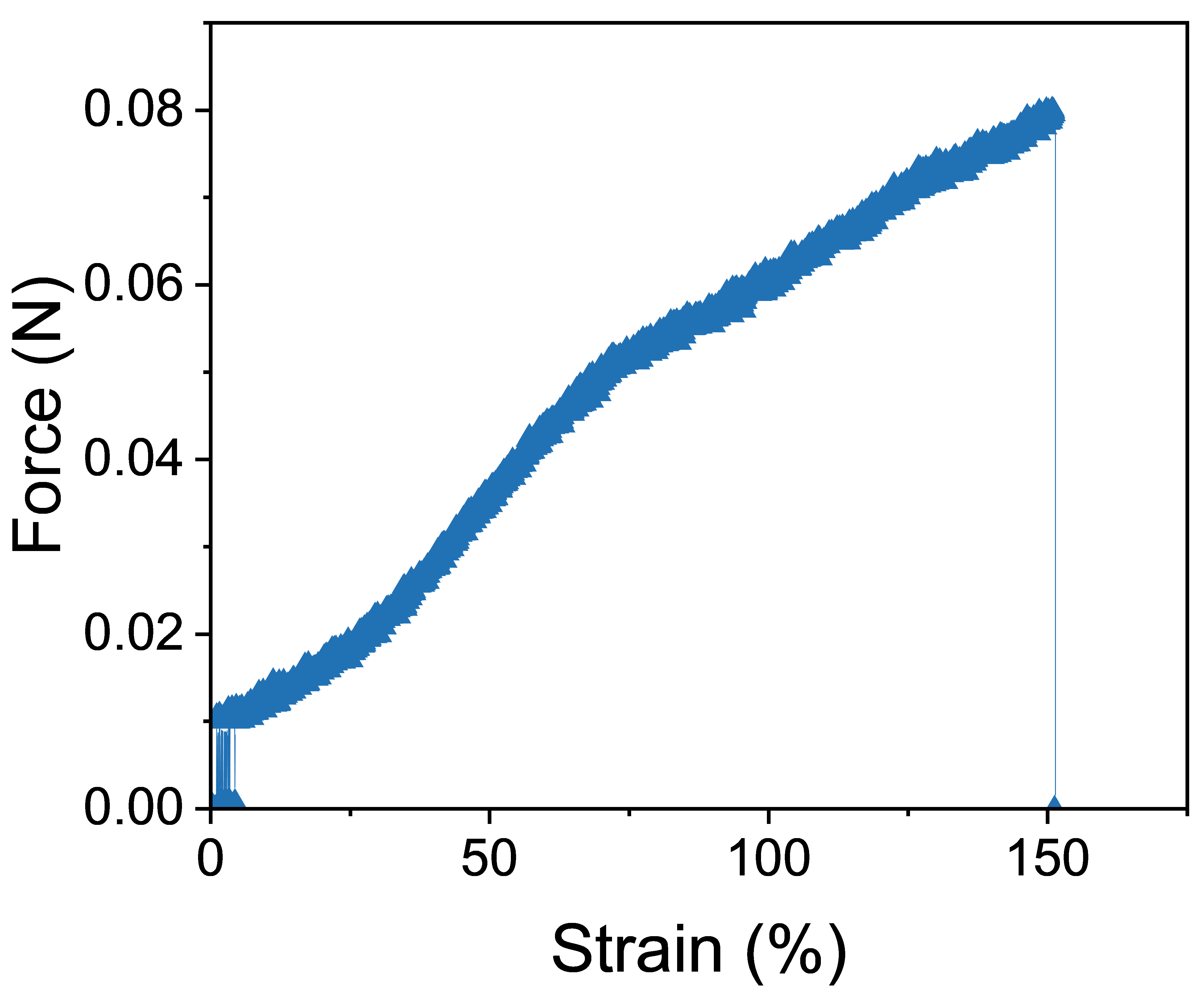


Figure S17**.** Mechanical curves of the pure TPTNa hydrogel with a notch.


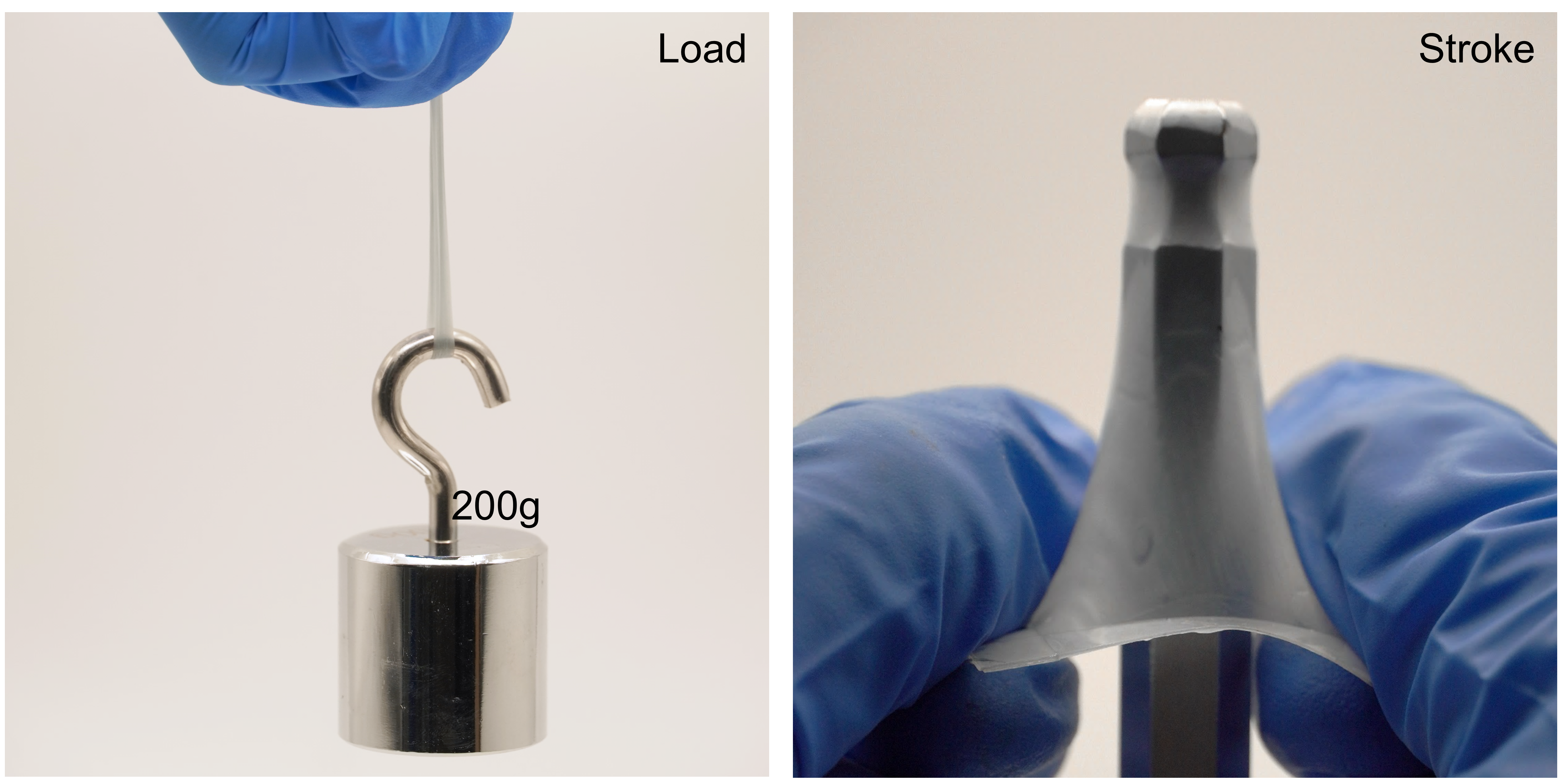


**Figure S18.** Photographs of a notched ultrathin nanomesh-reinforced hydrogel bearing a load and resisting puncture.


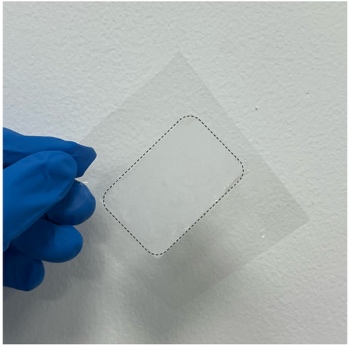


**Figure 19.** Cold-laminated ultrathin alginate hydrogels.


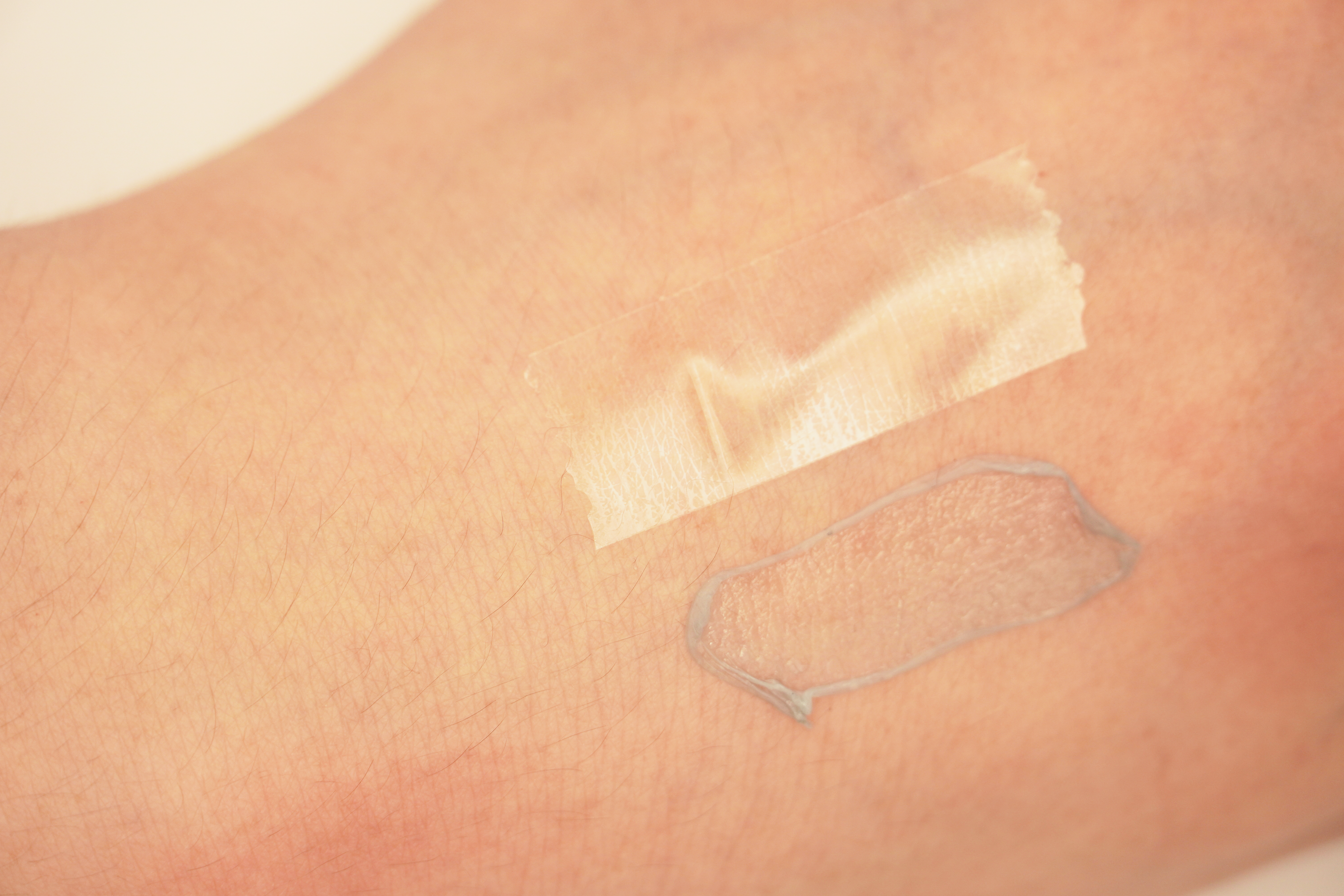


Figure S20**.** Digital image of skin covered by ultrathin nanomesh-reinforced hydrogel and PET adhesive tape.


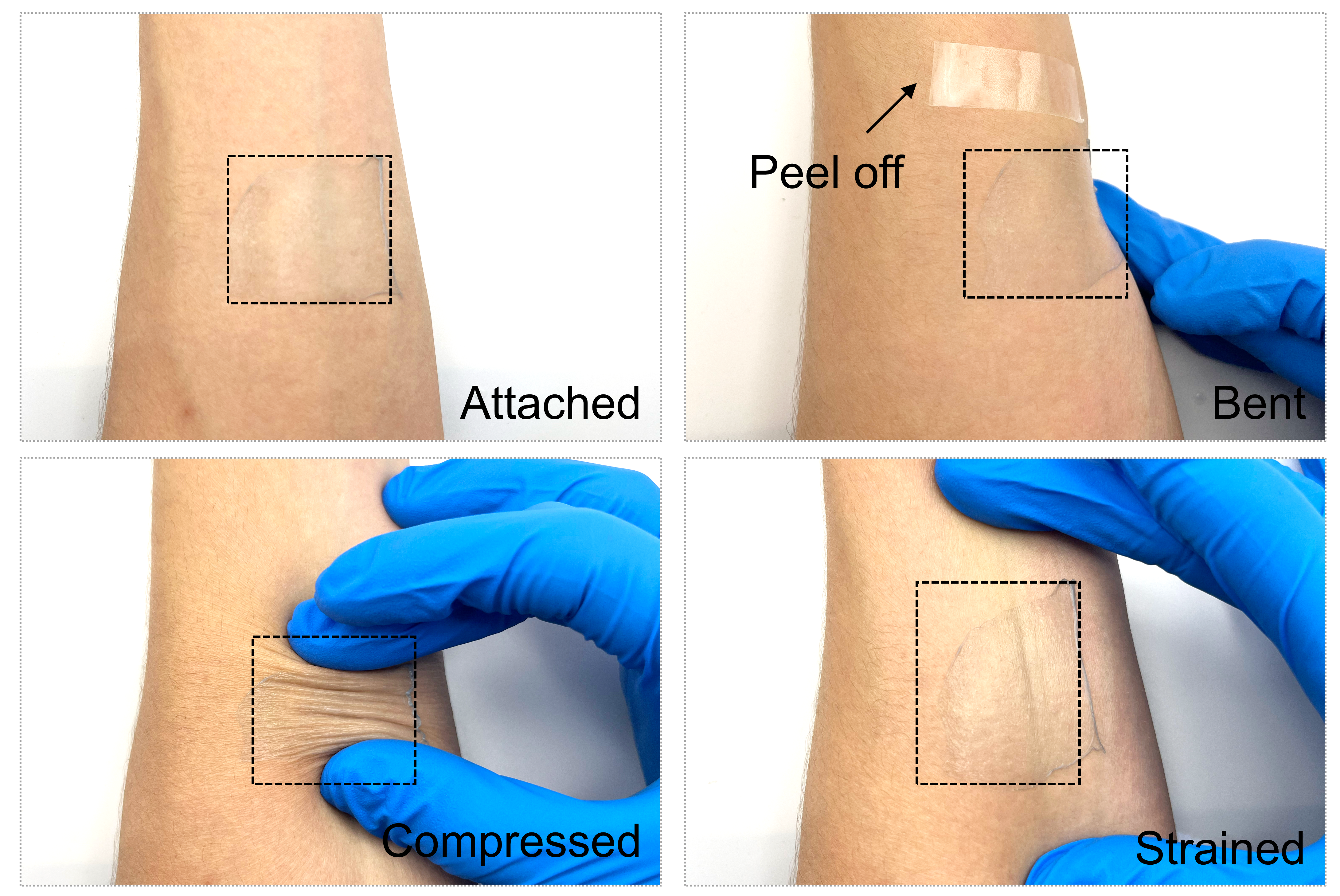


Figure S21**.** High skin conformability of the ultrathin nanomesh-reinforced hydrogel.


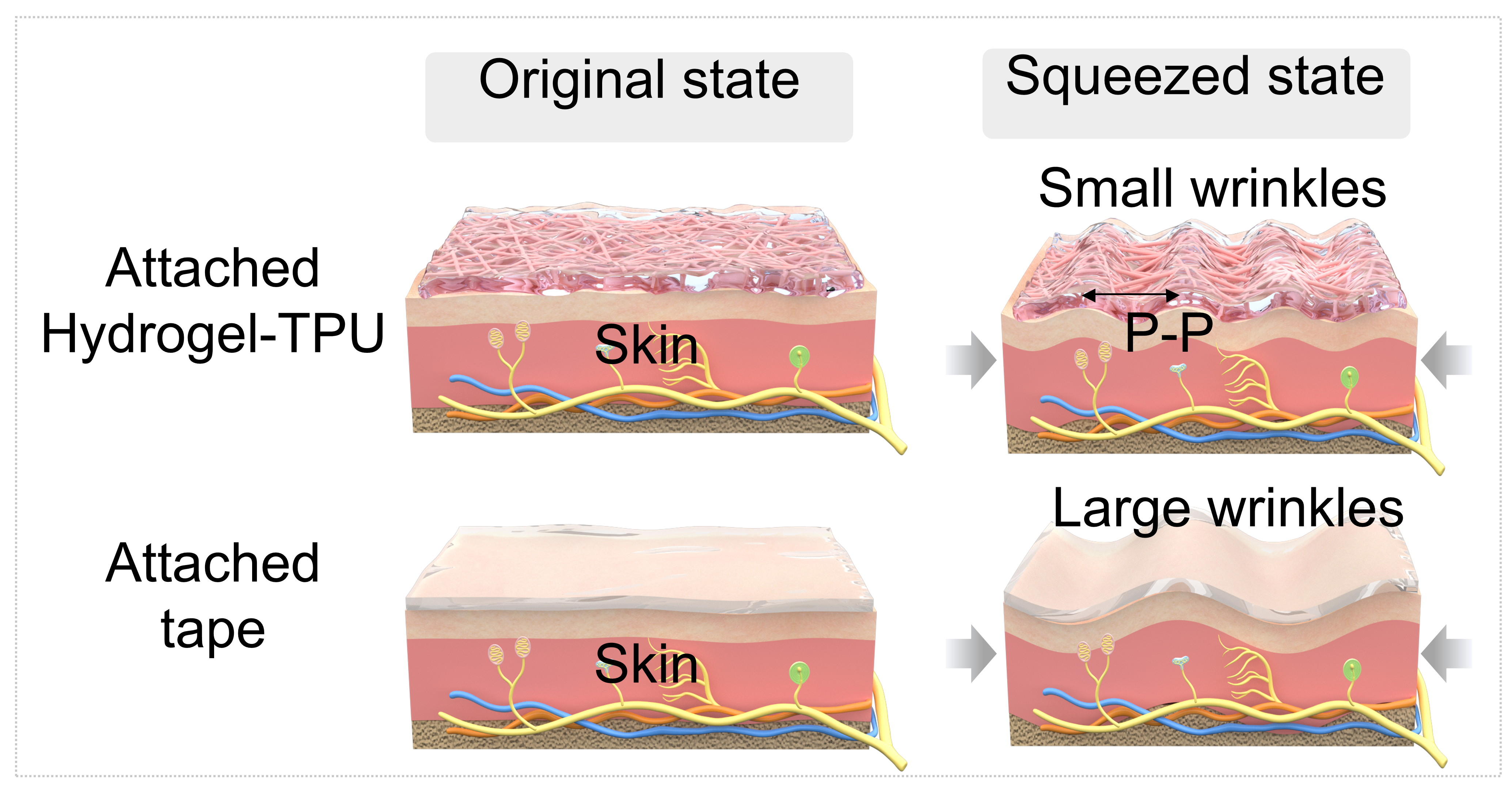


Figure S22**.** Illustration of the squeezing-induced wrinkle mechanism on skin with different surface coverings


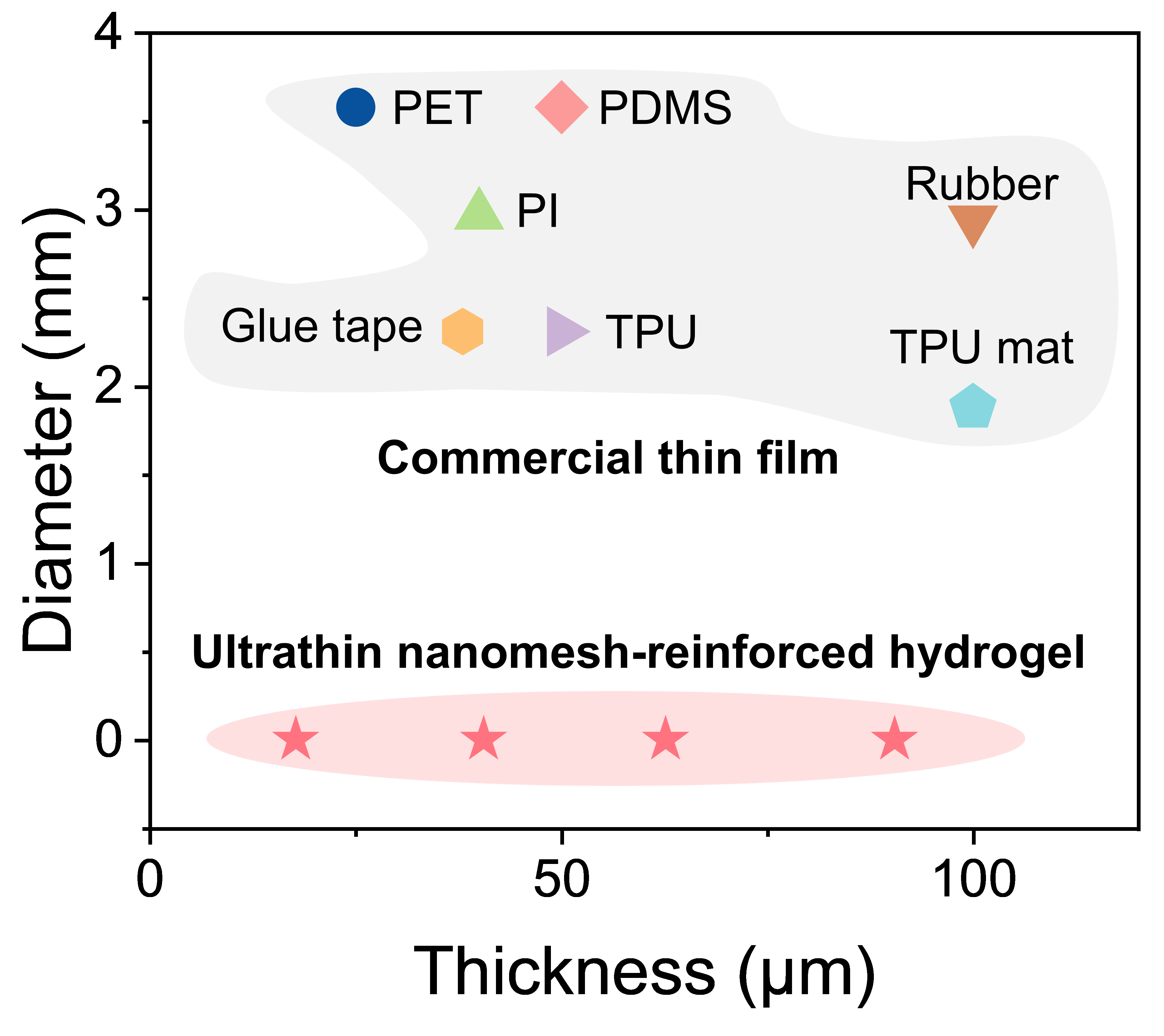


**Figure S23.** Comparison of bending circle diameters among different materials.


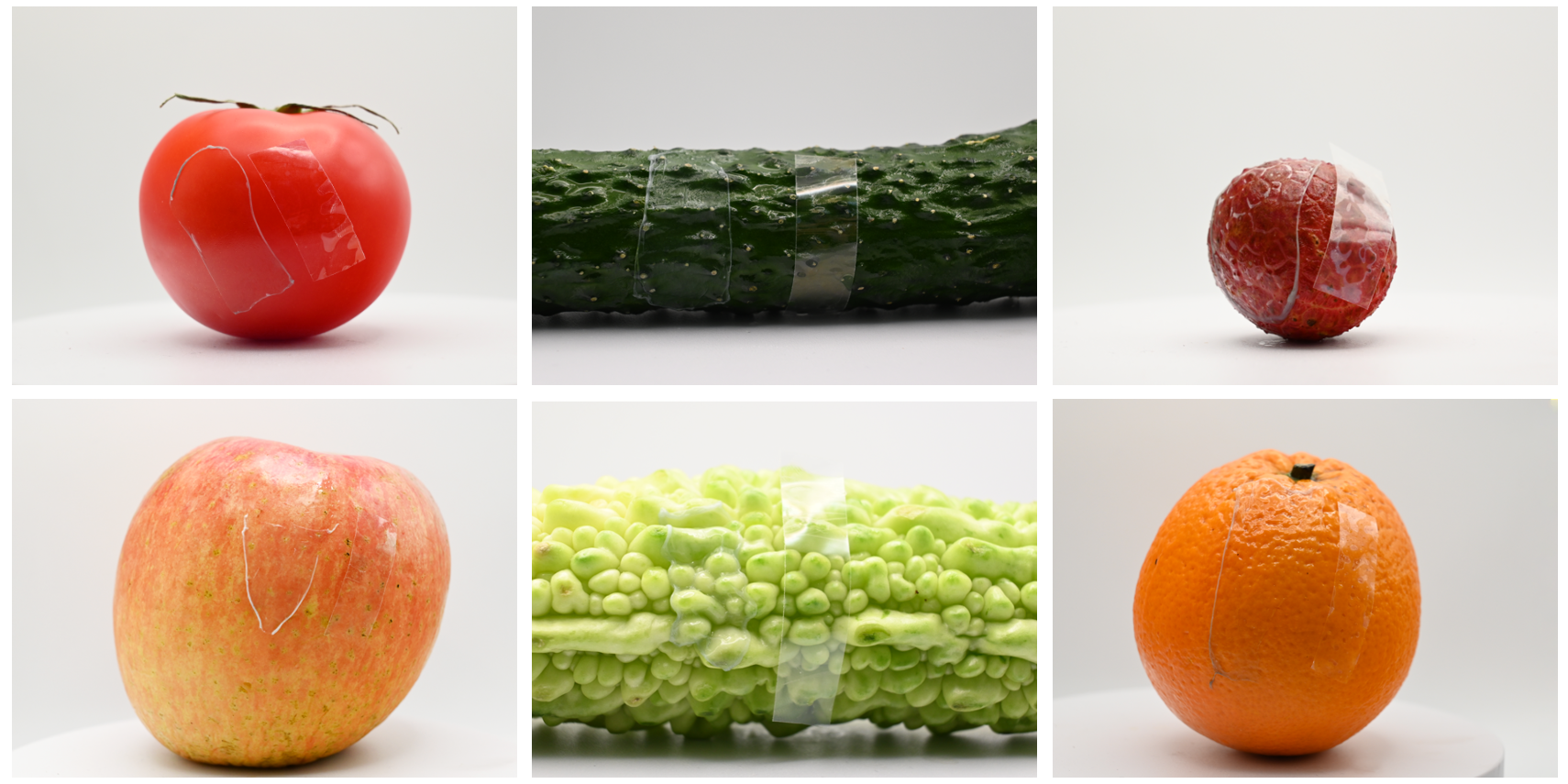


**Figure S24.** Digital photographs showing the conformal contact of ultrathin nanomesh-reinforced hydrogel (~40 μm thick) and PET adhesive tape on various objects with different surface textures, including tomato, cucumber, lychee, apple, bitter melon, and orange.

**Note S1. Mechanism of thermo-responsive adhesion**

The stimulating external field responsible for hydrogen-bond modulation in our hydrogel system is temperature. When the temperature is decreased, the hydrogen bonds are preferentially formed among the polymer chains of component P(AAc-co-NAGA), specifically between the amide groups of NAGA and the carboxyl groups of poly(acrylic acid), rather than between polymer chains and water molecules. This preferential polymer-polymer hydrogen bonding leads to a drastic reduction in the number of exposed polar groups at the hydrogel surface, thereby weakening its interfacial interactions with skin and resulting in a transition from strong adhesion to low adhesion.

To experimentally verify the temperature-dependent hydrogen-bond switching behavior, optical transparency tests were conducted. As the results shown in **Figure S23**, the hydrogel is transparent at 35 °C but becomes opaque when exposed to an ambient temperature of 20 °C. Upon returning to 35 °C, the hydrogel rapidly recovers its transparency, indicating its reversible temperature-responsive behavior. This optical transition is attributed to the formation of strong intermolecular hydrogen bonds at lower temperatures, which decreases the solubility of polymer chains and induces phase separation, leading to light scattering and opacity[26, 27]. At higher temperatures, polymer–polymer hydrogen bonds are dissociated, polymer–water hydrogen bonding is enhanced[28, 29], and the polymer chains become fully soluble, yielding a transparent hydrogel[30, 31]. This interpretation can be further supported by micro-morphological observations (**Figure S24**). At lower temperatures, the hydrogel exhibits a denser microstructure, reflecting the increased polymer association driven by formation of hydrogen bonding. Collectively, these optical and microstructural characterizations provide direct evidence that temperature effectively regulates hydrogen-bond interactions within the hydrogel through a reversible switching mechanism.


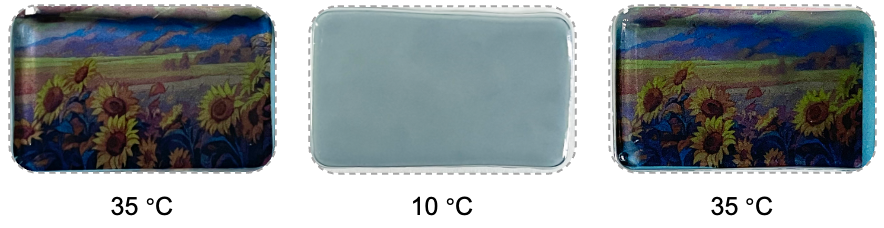


**Figure S25.** Photographs of the hydrogel showing its appearance at 35 °C, 10 °C, and after reheating to 35 °C


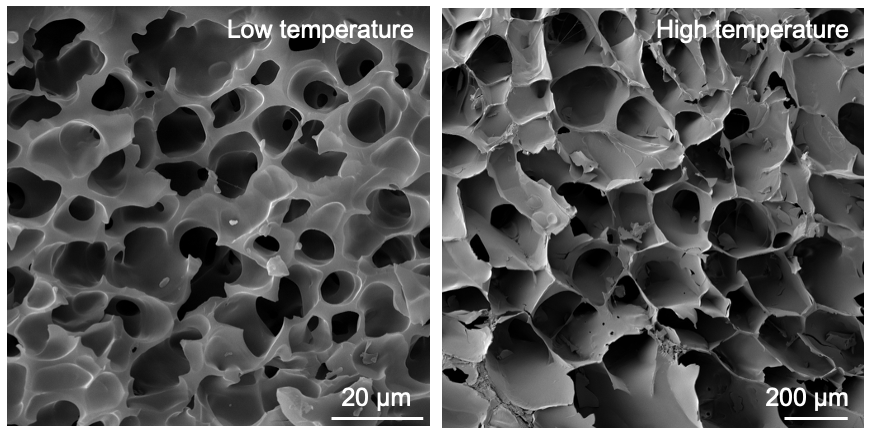


**Figure S26.** SEM of hydrogel under high and low temperatures.


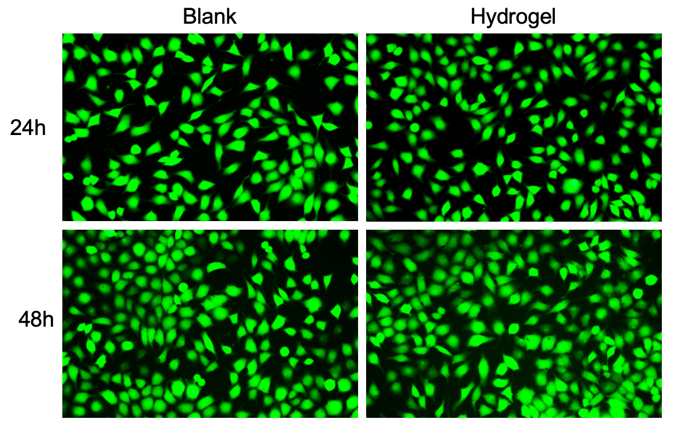


**Figure S27.** Biocompatibility evaluation of ultrathin nanomesh–reinforced hydrogels.

**Table S4.** Training configuration and hyperparameters of the dual-branch parallel deep neural network.

| Parameter | Value | Parameter | Value |
| --- | --- | --- | --- |
| Input size | 64 × 64 × 3 | Gradient threshold | 1 |
| Optimizer type | Adam | Mini-batch size | Default (MATLAB) |
| Maximum epochs | 500 | L2 regularization | 1 × 10⁻⁴ |
| Initial learning rate | 1 × 10⁻⁴ | Execution environment | Auto |
| Learning-rate decay | Yes | Loss function | Cross-entropy |

# Reference

1. Cheng, S., et al., *Ultrathin Hydrogel Films toward Breathable Skin-Integrated Electronics.* Advanced Materials, 2023. **35**(1): p. 2206793.

2. Zhang, Z., et al., *A 10-micrometer-thick nanomesh-reinforced gas-permeable hydrogel skin sensor for long-term electrophysiological monitoring.* Science Advances, 2024. **10**(2): p. eadj5389.

3. Wang, J., et al., *Fatigue-free artificial ionic skin toughened by self-healable elastic nanomesh.* Nature Communications, 2022. **13**(1): p. 4411.

4. Gao, Q., et al., *Biological Tissue-Inspired Ultrasoft, Ultrathin, and Mechanically Enhanced Microfiber Composite Hydrogel for Flexible Bioelectronics.* Nano-Micro Letters, 2023. **15**(1): p. 139.

5. Zhu, C., et al., *Breathable Ultrathin Film Sensors Based on Nanomesh Reinforced Anti-Dehydrating Organohydrogels for Motion Monitoring.* Advanced Functional Materials, 2024. **34**(52): p. 2411725.

6. Zhang, W., et al., *Fatigue of double-network hydrogels.* Engineering Fracture Mechanics, 2018. **187**: p. 74-93.

7. Liu, C., et al., *Tough hydrogels with rapid self-reinforcement.* Science, 2021. **372**(6546): p. 1078-1081.

8. Zhang, W., et al., *Fracture Toughness and Fatigue Threshold of Tough Hydrogels.* ACS Macro Letters, 2019. **8**(1): p. 17-23.

9. Kim, J., et al., *Fracture, fatigue, and friction of polymers in which entanglements greatly outnumber cross-links.* Science, 2021. **374**(6564): p. 212-216.

10. Wu, M., et al., *Stretchable freezing-tolerant triboelectric nanogenerator and strain sensor based on transparent, long-term stable, and highly conductive gelatin-based organohydrogel.* Nano Energy, 2022. **95**: p. 106967.

11. Liu, X., et al., *Graphene-Based Hydrogel Strain Sensors with Excellent Breathability for Motion Detection and Communication.* Macromolecular Materials and Engineering, 2022. **307**(8): p. 2200001.

12. Zhang, E., et al., *Fatigue fracture of nearly elastic hydrogels.* Soft Matter, 2018. **14**(18): p. 3563-3571.

13. You, L., et al., *Flexible porous Gelatin/Polypyrrole/Reduction graphene oxide organohydrogel for wearable electronics.* Journal of Colloid and Interface Science, 2022. **625**: p. 197-209.

14. Peng, H., et al., *Rapid Radiation Synthesis of a Flexible, Self-Healing, and Adhesive Ionogel with Environmental Tolerance for Multifunctional Strain Sensors.* ACS Applied Materials & Interfaces, 2023. **15**(44): p. 51763-51773.

15. Yan, S., et al., *Mechanically robust, transparent, conductive hydrogels based on hydrogen bonding, ionic coordination interactions and electrostatic interactions for light-curing 3D printing.* Chemical Engineering Journal, 2024. **486**: p. 150289.

16. Wang, C., et al., *Transdermal drug-delivery motion-sensing hydrogels for movement recovery caused by external injury.* Chemical Engineering Journal, 2024. **488**: p. 150998.

17. Hao, Y., et al., *A Stretchable, Breathable, And Self-Adhesive Electronic Skin with Multimodal Sensing Capabilities for Human-Centered Healthcare.* Advanced Functional Materials, 2023. **33**(44): p. 2303881.

18. Pi, M., et al., *Rapid Gelation of Tough and Anti-Swelling Hydrogels under Mild Conditions for Underwater Communication.* Advanced Functional Materials, 2023. **33**(1): p. 2210188.

19. Ye, T., et al., *Multifunctional visualized electronic skin based on a solvatochromic poly (ionic liquid) ionogel.* Chemical Engineering Journal, 2023. **477**: p. 147182.

20. Xiang, S., et al., *Multifunctional flexible sensors based on ionogel composed entirely of ionic liquid with long alkyl chains for enhancing mechanical properties.* Chemical Engineering Journal, 2022. **439**: p. 135644.

21. Xue, H., et al., *Hydrogel electrodes with conductive and substrate-adhesive layers for noninvasive long-term EEG acquisition.* Microsystems & Nanoengineering, 2023. **9**(1): p. 79.

22. Zhang, J., et al., *Development of Ultrathin, Breathable, Waterproof, and Durable Nanonet-Supported Ionogel Sensors for Electrophysiological Monitoring.* Advanced Functional Materials, 2025. **35**(8): p. 2415694.

23. Zhang, X.-F., et al., *Inorganic Salts Induce Thermally Reversible and Anti-Freezing Cellulose Hydrogels.* Angewandte Chemie International Edition, 2019. **58**(22): p. 7366-7370.

24. Wu, Y., et al., *Biomechanical Energy Harvesters Based on Ionic Conductive Organohydrogels via the Hofmeister Effect and Electrostatic Interaction.* ACS Nano, 2021. **15**(8): p. 13427-13435.

25. Zhou, J., et al., *Superior compressive and tensile bi-directional strain sensing capabilities achieved using liquid metal Hybrid-Hydrogels empowered by Machine learning algorithms.* Chemical Engineering Journal, 2024. **479**: p. 147790.

26. Liu, Y., et al., *Biomimetic Self-Deformation of Polymer Interpenetrating Network with Stretch-Induced Anisotropicity.* Chemistry of Materials, 2021. **33**(21): p. 8351-8359.

27. Liu, Z., et al., *Healable Strain Sensor Based on Tough and Eco-Friendly Biomimetic Supramolecular Waterborne Polyurethane.* ACS Applied Materials & Interfaces, 2022. **14**(4): p. 6016-6027.

28. Ge, S., et al., *Adjustable dual temperature-sensitive hydrogel based on a self-assembly cross-linking strategy with highly stretchable and healable properties.* Materials Horizons, 2021. **8**(4): p. 1189-1198.

29. Hua, L., et al., *Multiple-Responsive and Amphibious Hydrogel Actuator Based on Asymmetric UCST-Type Volume Phase Transition.* ACS Applied Materials & Interfaces, 2019. **11**(46): p. 43641-43648.

30. Beaudoin, G., et al., *Making Hydrophilic Polymers Thermoresponsive: The Upper Critical Solution Temperature of Copolymers of Acrylamide and Acrylic Acid.* Macromolecules, 2021. **54**(17): p. 7963-7969.

31. Roy, D., W.L.A. Brooks, and B.S. Sumerlin, *New directions in thermoresponsive polymers.* Chemical Society Reviews, 2013. **42**(17): p. 7214-7243.
